# Immunization with a *Trypanosoma cruzi* cyclophilin-19 deletion mutant protects against acute Chagas disease in mice

**DOI:** 10.1038/s41541-023-00647-5

**Published:** 2023-04-25

**Authors:** Bijay Kumar Jha, Sanjay Varikuti, Chaitenya Verma, Rahul Shivahare, Nicholas Bishop, Gregory P. Dos Santos, Jacquelyn McDonald, Aakash Sur, Peter J. Myler, Sergio Schenkman, Abhay R. Satoskar, Bradford S. McGwire

**Affiliations:** 1 Division of Infectious Diseases, Department of Medicine, Wexner Medical Center, The Ohio State University, Columbus, OH USA; 2 Departments of Pathology and Microbiology, Wexner Medical Center, The Ohio State University, Columbus, OH USA; 3 Department of Microbiology, Immunology and Parasitology, Federal University of Sao Paulo, Sao Paulo, Brazil; 4 Center for Global Infectious Disease Research, Seattle Children’s Research Institute, Seattle, WA USA; 5 Department of Biomedical Informatics and Medical Education, University of Washington, Seattle, WA USA; 6 Department of Pediatrics, University of Washington, Seattle, WA USA; 7 Department of Global Health, University of Washington, Seattle, WA USA

**Keywords:** Live attenuated vaccines, Parasitic infection, Cellular microbiology, Parasitic infection, Infection

## Abstract

Human infection with the protozoan parasite *Trypanosoma cruzi* causes Chagas disease for which there are no prophylactic vaccines. Cyclophilin 19 is a secreted cis-trans peptidyl isomerase expressed in all life stages of *Trypanosoma cruzi*. This protein in the insect stage leads to the inactivation of insect anti-parasitic peptides and parasite transformation whereas in the intracellular amastigotes it participates in generating ROS promoting the growth of parasites. We have generated a parasite mutant with depleted expression of Cyp19 by removal of 2 of 3 genes encoding this protein using double allelic homologous recombination. The mutant parasite line failed to replicate when inoculated into host cells in vitro or in mice indicating that Cyp19 is critical for infectivity. The mutant parasite line also fails to replicate in or cause clinical disease in immuno-deficient mice further validating their lack of virulence. Repeated inoculation of mutant parasites into immuno-competent mice elicits parasite-specific trypanolytic antibodies and a Th-1 biased immune response and challenge of mutant immunized mice with virulent wild-type parasites is 100% effective at preventing death from acute disease. These results suggest that parasite Cyp19 may be candidate for small molecule drug targeting and that the mutant parasite line may warrant further immunization studies for prevention of Chagas disease.

## Introduction

*Trypanosoma cruzi* is the etiologic agent of Chagas disease, which is endemic in Mexico, Central and South America, and in regions within the lower United States, particularly in Arizona, New Mexico, and Texas^1–3^. The infection is transmitted in multiple ways, chiefly by the feeding of hematophagous triatomine insects on mammalian hosts^2^. Humans are incidental hosts and those living in rural areas are at the greatest risk where poor housing conditions lead to the entrance and feeding of triatomines^4^. Feeding leads to the deposition of fecal material containing infective metacyclic trypomastigote forms that contaminate mucosa membranes and insect feeding sites^3^. Metacyclics bind to and invade host cells wherein they transform into and multiply in the host cell cytosol as intracellular amastigotes. Amastigotes transform thereafter into motile trypomastigotes that exit host cells, these parasites then circulate through the body to further infect cells in a variety of target organs. Long-term infection of tissue, particularly cardiac myocytes and gastrointestinal smooth muscle, causes the sequelae of chronic Chagas disease^5^. Additional modes of infection are by oral ingestion of parasite-infected food or liquids, trans-placental maternal-fetal transmission, and through transplant of infected blood and tissues to non-infected recipients^6–9^. Importantly, the global transmigration of chronically infected hosts leads to the diagnosis of Chagas disease in countries that are not endemic for Chagas. Approximately 20–30% of chronically infected individuals develop chronic symptoms, which include Chagas-associated cardiomyopathy, due to chronic inflammation of cardiac tissue from direct parasite damage and molecular mimicry^10,11^. Chagas disease is the leading cause of heart failure in Latin America. Mega-organ syndromes of the esophagus and large intestine arise from denervation of the gastrointestinal tract due to direct or indirect damage of the smooth muscle^12,13^.

Upon initial infection, there is limited exposure of the immune system to parasite antigens, since the inoculum is small and parasites rapidly become intracellular. Parasites replicate intracellularly and exit host cells in larger numbers resulting in immune activation^14^. The immune response to *T. cruzi* is complex, but involves the induction of both Th1 and Th2 responses. Th-1 responses are signaled by the production of IL-12 leading to IFN-γ and TNF-α production by macrophages. Activated inflammatory cells produce IL-1, -6, and -18 and are poised to kill intracellular parasites^15,16^. Anti-inflammatory Th2 responses are also activated in order to control over-aggressive immune activation and involve IL-4 and -10 production^17,18^. Additionally, CD8+ lymphocytes and anti-parasite IgG are produced which limit the increase in parasite load^19^. Chronic smoldering infection ensues after several years due to several factors eventually leading to chronic sequelae of Chagas disease in about one-third of infected patients. It is unclear why only a subset of infected individuals develop these chronic manifestations, but this is likely due to the complex interplay of host genetic and parasite factors. There are no vaccines available for prevention of Chagas disease and treatment is limited to two drugs, which have significant side effects.

Several Chagas vaccines have been described including those based on recombinant protein subunits^20–22^, naked plasmid DNA-protein expressing constructs^23^, peptides^24^, adenoviral and *Salmonella* vector systems for expression of parasite proteins as well as several live attenuated *T. cruzi* vaccines^25–28^. Live vaccine strains include those attenuated by long-term in vitro cultivation^29^, chemical treatment and those containing engineered mutations^30–32^. These are either mono-allelic or bi-allelic deletion mutants of single copy genes in several *T. cruzi* parasite strains. Immunization of a mono-allelic deletion mutant in calreticulin, was able to reduce parasitemia, mortality and tissue inflammation when challenged with highly virulent parasites. Interestingly, the mutant did not generate demonstrable anti-parasite antibody and protection was thought to be due to induction of cellular immunity^33^. A bi-allelic mutant in the flagellar protein gp72, was shown to have limited persistence in infected immuno-competent mice and induced anti-parasite antibodies in a minority of the animals and was able to protect them from lethal challenge with highly virulent parasites. The level of protection of this mutant was similar to that of the attenuated parental wild type suggesting that the reduced level of infectivity of the mutant was independent of the capacity to protect animals from challenge^34^. Similarly, a mono-allelic deletion mutants in the dhfr-ts gene, defective in growth in vitro and in vivo, was able to induce long-term protection of mice similar to its attenuated parental wild-type strain, even with reduced level of CD8+ cells compared to the wild type immunized animals^31^. Deletion mutants in 3 out of 4 genes encoding Enoyl-coenzyme A hydratases were found to be effective in protection following oral administration followed by footpad challenge in a detection system using fluorescent-parasites. Protection in this system correlated with the high levels of activated CD8+ cells^35^. Immunization with an attenuated mutant lacking one of two gene copies encoding calmodulin-ubiquitin was also able to significantly reduce parasitemia on subsequent challenge with wild-type parasites^36^. A mutant in the protein LYT1, has attenuated infectivity in mice and pre-inoculated with this mutant had reduced parasitemia, splenic parasite indexes, and tissue parasite burden when challenged with virulent parasites^37^.

The major advantage of bi-allelic deletion attenuated mutants is the lack of reversion potential through repair of the mutated allele by the intact allele or duplication events. Potential theoretical advantages of such live vaccine candidates include: exposure of the immune system to an entire range of parasite antigens to help bolster a more effective immune response, infection of the host through the natural route to target the same host cell types as wild-type parasites and creation a persistent non-lethal infection leading to continuous antigen exposure for long-lasting immunity. The potential downsides of such vaccines are the potential for loss of attenuation and persistence of infection leading to chronic immune stimulation and tissue damage.

Our recent studies have focused on parasite cyclophilin 19, an enzyme that catalyzes cis-trans peptidyl-prolyl isomerization of proteins (PPIases). This protein is secreted by epimastigotes and is important in the neutralization of parasiticidal proline-containing insect anti-microbial peptides^38^. As a group, PPIases are expressed by organisms from all Kingdoms where they serve multiple functions including acting as a protein chaperones (folding of nascent proteins and re-folding of aggregated proteins), in ROS production and scavenging^39^, gene expression and RISC complex formation and miRNA regulation^40,41^. Cyp19 is the only *T. cruzi* PPIase that has been reported to be secreted^38,42–44^ which portends a role in interacting with extracellular substrates. We recently reported that Cyp19 is expressed by and released extracellularly by all life-stages of the parasite^45^ suggesting that in each parasite stage it may function to engage and modify different host target proteins. Our recent data also indicates that secreted Cyp19 by intracellular amastigotes induces intracellular ROS production in host cells, which is critical for amastigote replication^45^. In order to determine the role of cyclophilin 19 in parasite virulence and pathogenesis, we employed a homologous recombination gene knock-out strategy to remove two of three cyclophilin 19 genes from parasites. This parasite mutant is unable to produce amastigotes or trypomastigotes in host cells in vitro nor are they able to grow in, or produce acute disease mice. Repeated inoculation of mice with these mutants leads to increasing levels of parasite-specific immune response that is 100% effective at preventing death from acute Chagas disease upon challenge with wild-type parasites. The mutant strain does not cause acute disease in immuno-deficient mice strains and dexamethasone treatment of mice inoculated with the mutant strain did not develop clinical disease or the emergence of parasites indicating that the mutant line is safe in immune-suppressed host conditions. Lastly, a single dose of this attenuated parasite line led to complete protection upon challenge with highly virulent wild-type parasites. Overall, this data is proof of principle that this attenuated mutant line is effective at preventing acute Chagas disease in the mouse model.

## Results

### Generation of a Cyp19 knock-out parasite line

**Fig. 1:**
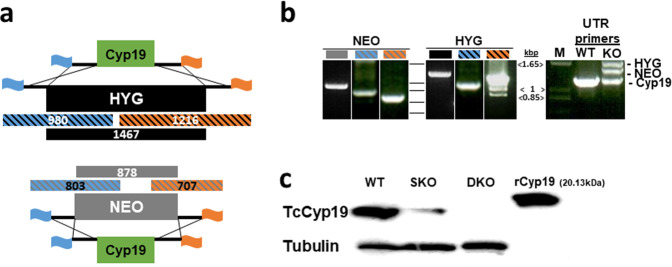
DNA and protein analysis of Cyp19 knock-out parasites. **a** Strategy of Cyp19 gene knock-out using double allelic replacement with genes for neomycin phosphotransferase (NEO) and hygromycin phosphotransferase (HYG) genes. Predicted PCR fragment sizes as indicated (in bps). Right and left chromosomal flanking regions depicted by orange and blue flags. **b** PCR analysis of NEO and HYG integration of purified chromosomal parasite DNA showing each pair of primers. 5′- and 3′-UTR primers were used to confirm presence of NEO and HYG gene integration, but also identified presence of a 3rd copy of Cyp19 (far right panel). **c** Western blot analysis for Cyp19 protein in wild type (WT) SKO-NEO (SKO) and DKO cells. α-tubulin analysis was performed in duplicate blots as a loading control for each cell line (as shown). rCyp19 is purified His-tagged Cyp19, as a control. Source data for PCR and western blotting is found in Supplementary Figs. 14 and 15.

Since the published genome sequence of *T. cruzi* (*CL Brener* strain)^46^ indicated that cyclophilin 19 is expressed from a single copy gene^43,47^, we opted to create a Cyp19 depletion mutant using double allelic gene replacement (Fig. 1a). Plasmid constructs were engineered to contain 5′- and 3′-UTRs of Cyp19 flanking sequences surrounding drug resistance genes for neomycin- and hygromycin phospho-transferases (NEO and HYG, respectively). These were amplified by PCR, cloned into pET15b plasmids, linearized and transfected separately into Brazil strain epimastigotes and selected using G418 and hygromycin, respectively. Drug-resistant parasites were cloned using limiting dilution and examined for appropriate chromosomal integration of drug-resistant constructs using PCR. A second allelic ablation was performed from a G418-resistant clone with another round of transfection using a HYG-resistance marker containing construct. After double drug selection and limited dilution cloning, parasites were analyzed by PCR for integration of transgenes, Cyp19, and flanking sequences (Fig. 1b). PCR analysis of DNA from wild-type and the DKO parasites across the entire region of the Cyp19 loci using primers from the 5′- and 3′-UTRs unexpectedly found an additional intact Cyp19 gene (Fig. 1b, right panel). Western blot analysis of doubly resistant parasites confirmed depletion of Cyp19 protein expression (Fig. 1c). Cyp19 was not detected in the mutant cells despite the presence of a lingering Cyp19 gene, suggested either that the protein is not expressed, expressed at low level, or that it is an isoform that was significantly different from that expressed by genes in the other two alleles and not recognized by our antiserum. In this paper, SKO and DKO refer to parasite lines harboring the removal of one or two Cyp19 genes, respectively.

### Whole nuclear genome sequencing of wild-type and Cyp19 knock-out strains

In order to verify the removal of Cyp19 genes and integration of drug markers and to understand the origin of the lingering Cyp19 gene copy we performed whole genome sequencing of several knock-out clones (denoted as SKO-NEO and -HYG, DKO-D0, -D11, -D12 in Supplementary Figs. 1–4) and two WT parental strains. DKO-D0 is the parasite line used in the vaccination studies in this paper. DKO lines D11 and D12 were recloned from the DKO-D0 cloned line more than one year after continued growth in culture. The WT strains included parental WT strain used for the knock-out (termed WT-att), and an additional virulent WT strain (termed WT-vir) derived from WT-att by multi-passage through immuno-deficient STAT 4^−/−^ mice. Recently the genome sequence of Brazil strain A4^46^ was published which we used as a reference to compare the genome sequences. This analysis confirmed the absence of two Cyp19 genes and replacement with NEO and HYG as expected (Supplementary Fig. 1). We indeed found that there was trisomy of the central portion of chromosome 1, containing one additional copy of the Cyp19 gene in both the WT strains (Supplementary Figs. 1–3). This indicated that Cyp19 trisomy pre-existed in both WT parasite lines before transfection and was not a result of compensation for loss of two Cyp19 gene copies. Interestingly, left segment of chromosome 1 appeared to be diploid in WT and SKO lines, but triploid in the DKO lines, whereas the right segment appeared to be diploid in all of the cell lines. This suggests that each segment could represent a fusion of different chromosomes, or that chromosome 1 had undergone fragmentation during duplication events. The presence of trisomy of the left segment in only the DKO clones may indicate some level of functional compensation for loss of the two Cyp19 copies in those clones despite the absence of Cyp19 genes in this region. The predicted amino acid sequence of the last Cyp19 gene present in the central region is identical to replaced copies, negating the notion that it could be a different isoform. There is considerable aneuploidy between the cell lines and there are increased copy number of several putative cyclophilin-like genes in the DKO lines as a result of trisomy of chromosomes 17 and 22 (Supplementary Fig. 4). It is unclear whether these changes are also a result of functional compensation for loss of two of the three Cyp19 genes in the DKO. The overall genomic features of the DKO lines are highly stable inasmuch as DKO-D0 and -D11, -12 are unchanged after one year of continuous growth in culture (without drug pressure) and there has been no repair of the inactivated Cyp19 alleles containing NEO or HYG genes by the remaining intact gene copy. The loss of two Cyp19 genes results in stable attenuation of pathogenesis of the DKO lines (see sections below). We have spent a considerable effort in trying to remove the last Cyp19 copy using a 3rd allelic replacement without success. However, during completion of the studies reported here, we have successfully inactivated expression from the remaining Cyp19 gene, as well as NEO and HYG genes from the DKO line using triple stop codon insertions using CRISPR/Cas9 technology. Studies with these new lines are ongoing currently.

### Parasite growth and infection studies in vitro

**Fig. 2:**
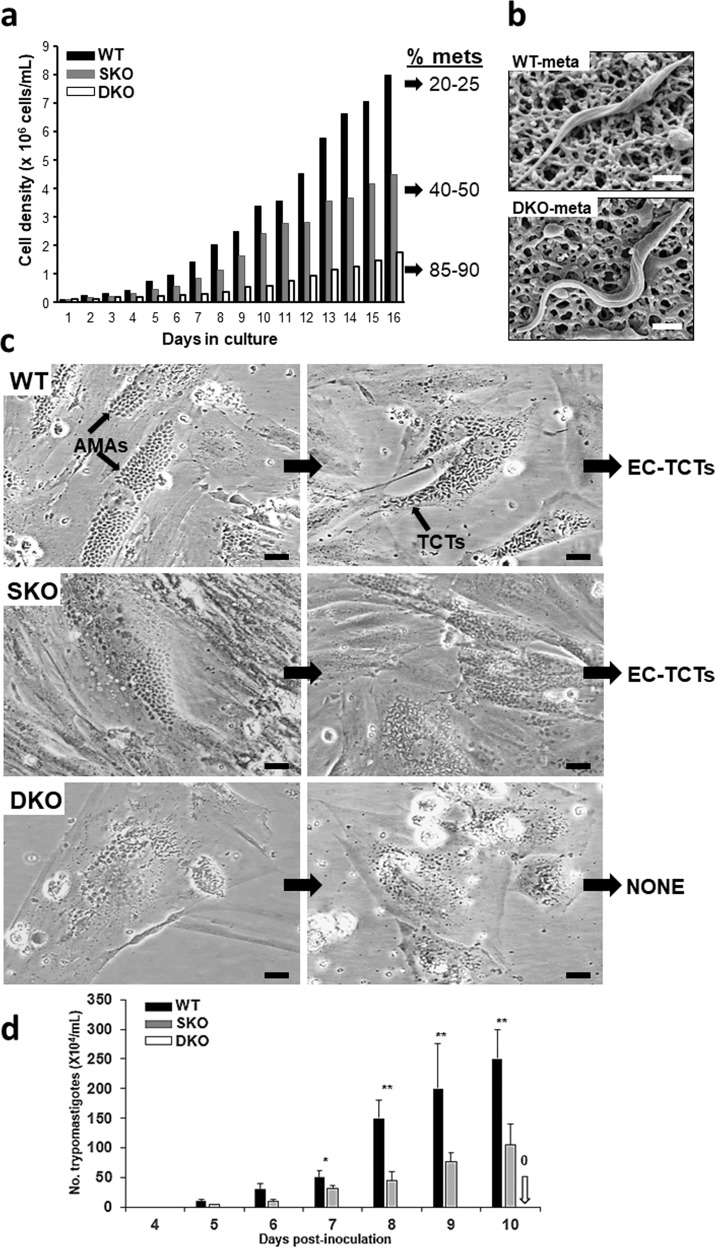
Growth and infection analysis of mutant *T. cruzi* cell lines. **a** Growth of cell lines in culture. Comparison of wild type (WT), single knock-out (SKO), and double knock-out (DKO) lines demonstrated that SKO and DKO lines have diminished growth in culture with higher rates of conversion to metacyclic trypomastigotes. **b** Metacyclic forms of WT and DKO lines are morphologically similar in scanning EM analysis. **c** In vitro incubation of H9C2 cells (rat heart myoblasts) with isolated metacyclic trypomastigotes of WT, SKO, and DKO cell lines after 3 days (left column) and 6 days (right column) post-infection. WT and SKO infect host cells, develop into amastigotes that transform into and emerge as extracellular tissue culture trypomastigotes (EC-TCTs). DKOs do not replicate as amastigotes and do not produce EC-TCTs. **d** Quantitation of production of EC-TCTs by the various parasite lines. Infections were performed in triplicate and each was counted twice per sample on days 4–10 post-inoculation. The white arrow indicates the lack of TCT production for the DKO line, which remained undetectable throughout the incubation period. Student t-test was used. *P*-values: *<0.05, **<0.001. Scale bars = 2.5 μm in panel (**b**), 20 μm in panel (**c**).

Epimastigotes of the Cyp19 DKO mutants grew slower and reached peak densities that were significantly lower than their wild-type controls (Fig. 2a). The proportion of metacyclic forms that arose spontaneously in culture appeared earlier in the course of growth in culture and were significantly more abundant than in wild-type parasite cultures, reaching approximately 85–90% of the population (Fig. 2a). Single knock-out (SKO) parasites reached approximately half the peak density of WT parasites and produced about 35–50% metacyclic forms during growth in culture (Fig. 2a). The metacyclics of Cyp19 DKO parasites are resistant to complement-mediated killing using normal human serum, as are those of wild-type parasites, and had morphology similar to wild-type metacyclic forms (Fig. 2b). We suspect that diminished Cyp19 expression in the knock-out parasite lines leads to faster differentiation of epimastigotes into metacyclic trypomastigotes resulting in the higher proportion of metacyclics in the overall culture. Since metacyclic parasites do not replicate, this results in reduced overall density of mutant parasites in culture.

In order to test the ability of SKO and DKO parasite lines to infect we used a rat heart myoblast (RHM) cell line (H9C2), our prototype line for in vitro assessment of parasite infection, and incubated them with isolated metacyclics of DKO, SKO, and WT parasite lines. Infection of RHMs with WT parasites results in the development of intracellular amastigotes by 2–3 days post-infection that increase in number, eventually transforming into motile tissue-culture trypomastigotes (TCTs) which then exit host cells (Fig. 2c, upper panels and d). The course of infection of RHMs with SKO parasites is similar to that of WT parasites, although they appear to grow slower intra-cytoplasmically, but eventually reach similar densities and transform into TCTs that exit the cell (Fig. 2c, middle panels). Infection of RHMs with DKO parasites results in the production of amastigote-like bodies, which fail to replicate intracellularly and eventually degenerate (Fig. 2c, lower panels). RHMs incubated with DKO parasites fail to give rise to demonstrable TCTs (Fig. 2c, d). We have not observed amastigote replication or TCTs forms produced over prolonged observations of RHMs cultures incubated with DKO parasites for 1–2 months. We have repeated infectivity studies with the DKO line at least 10 times with the same results. The additional DKO clones, D-11 and D-12, obtained by recloning the DKO line after one year on continuous culture, were also repeatedly tested for their ability to replicate in H9C2 cells. Neither parasite line was able to replicate within RHMs or produce TCTs, as we have seen with the original DKO parasite line (DKO-D0). We have also used fluorescent microscopy of CFSE-labeled WT and DKO lines incubated with RHMs and have found that the DKO parasites indeed persist in host cells but do not increase in number, confirming that once they gain entry, these parasites appear blocked in their ability to replicate as amastigotes or differentiate to TCTs (Supplementary Fig. 5). We also compared the ability of WT and DKO parasites to cause productive infection in phagocytic RAW cells (Supplementary Fig. 6). As expected, infection of these cells with WT metacyclics led to the development of amastigotes and formation and release of extracellular trypomastigotes (Supplementary Fig. 6, left panel). In contrast, infection of cells with DKO metacyclics do not lead to the development of replicating intracellular amastigotes or the production of extracellular trypomastigotes.

### Infection studies in mice

**Fig. 3:**
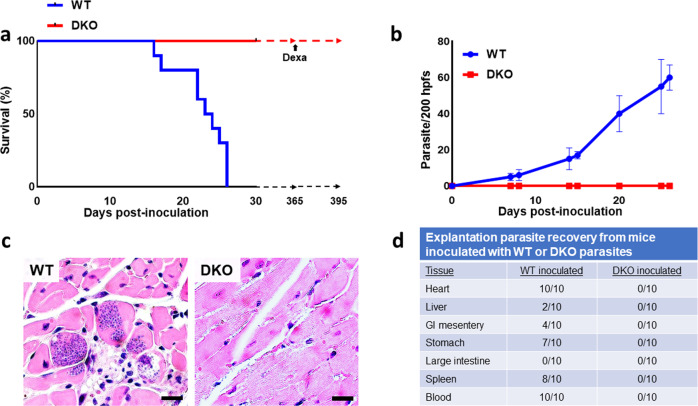
Inoculation of mice with *T. cruzi* cell lines. **a** Survival curve of mice inoculated with WT and DKO cell-culture-derived metacyclic trypomastigotes (10^5^ intraperitoneally per mouse. *n* = 20) demonstrate that WT parasites kill all mice within ~21 days, inoculation of mice with DKO parasites does not result in clinical infection up to one-year post-inoculation, in all groups. DKO-inoculated animals (2 experiments of 5 animals each) which had been observed for one year, treated with dexamethasone (Dexa) did not develop clinical disease, alter survival or cause the emergence of parasites from blood or tissues in 100% of the animals (Supplementary Fig. 13). **b** Parasitemia of mice inoculated with WT or DKO parasites (*n* = 10 mice per assay). **c** Histopathologic analysis of heart sections of mice inoculated with WT (left) taken from mice at or near the time of death at 21 days or DKO parasites (right) harvested at 6 months post-infection. **d** Explantation of indicated tissues in parasite culture medium from mice either at or near time of death (for WT-inoculated animals) or 6 months (for DKO-inoculated animals) post-infection. This is a compilation of data from two separate experiments with comparable results (5 mice per group in each experiment, *n* = 10). Student t-test was used for parasitemia. *P* < 0.05 in panel **b**. The log-rank (Mantel-Cox) test was used for Kaplan–Meier survival curves.

In order to test the effect of the loss of Cyp19 on parasite virulence in animals, we used immuno-competent AJ mice (males 4–6 weeks of age, 5 animals per group) inoculated with cell-culture-derived metacyclics of either WT or DKO lines (10^5^ parasites per intraperitoneal inoculation). Control infections with WT parasites consistently resulted in the onset of clinical infection in all animals (shivering, ruffled coat, huddling behavior, diminished appetite and activity) within 1–1.5 weeks and death within 16–25 days post-infection (Fig. 3a). The peak density of parasitemia in these mice just prior to death or sacrifice were between 45–70 parasites per 200 high power fields (100X) (hfps/mouse). Inoculation of animals with DKO parasites result in neither the development of clinical disease in 100% of the animals and all of the mice survived infection (Fig. 3a). Over the course of two separate experiments (5 animals per group/experiment), we did not observe clinical signs of infection in any of the animals and all mice survived and remained healthy up to 15 weeks post-infection with DKO parasites. Over the course of one year of observation, all of the DKO-inoculated mice showed no clinical signs of infection and remained healthy. We could not detect parasitemia in weekly microscopic analysis of the blood from DKO-inoculated animals. Analysis of tissues (liver, spleen, heart, GI mesentery, stomach, and large intestine) from these animals using both tissue explantation culture and histopathology demonstrated abundant parasites in most tissues from WT-inoculated animals whereas we could not find parasites in DKO-inoculated animals (Fig. 3b, c). *T. cruzi*-specific PCR analysis of cardiac tissue from DKO-inoculated animals did produce signals consistent with the presence of parasite DNA (Supplementary Fig. 7) at 15 weeks post-infection. In addition, PCR reactions using NEO-specific primers indicated the presence of DKO DNA. In the absence of culturable parasites from tissue, this likely represents the presence of latent non-replicating intracellular parasites as seen in in vitro infections. Total RNA purified from the tissues of animals inoculated with WT- and DKO-parasites were subjected to RT-PCR analysis to order to assess whether parasites persisting in tissue were undergoing gene expression. Only tissue from the WT-inoculated animals gave rise to detectable mRNA expression from the *T. cruzi* specific microsatellite sequence, TCZ, whereas tissue from DKO-inoculated animals did not (Supplementary Fig. 8).

Mice (5 mice per group, in two separate experiments) inoculated with DKO parasites were treated with dexamethasone (5 mg/kg) one-year post-inoculation in order to test whether induction of immunosuppression would induce outgrowth of latent parasites or result in clinical disease. Despite thirty days of treatment of DKO-inoculated animals with dexamethasone, all of these mice remained clinically healthy. Further, parasites were undetectable in all animals when examined by explantation or histopathologic analyses of cardiac tissue (Supplementary Fig. 13).

### Cyp19-depleted parasites do not cause clinical infection in immuno-deficient mouse strains

**Fig. 4:**
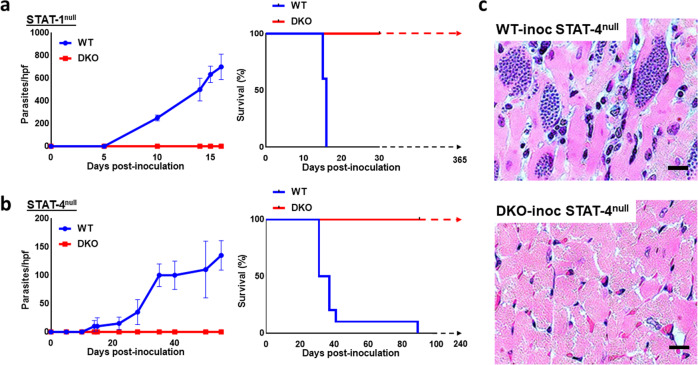
Inoculation of WT and DKO parasite lines in immuno-deficient mouse strains. Both STAT-1^null^ (**a**) and STAT-4^null^ (**b**) mice were inoculated with WT and DKO cell-culture-derived metacyclic trypomastigotes (10^5^ parasite per IP inoculation per mouse) and followed for parasitemia (left panels), survival (middle panels). Data demonstrates that WT parasites increase in the blood over the course of infection until death between 15–16 days (for STAT-1^null^ animals) or between 31–89 days (in STAT-4^null^ animals). Inoculation of either mouse strain with DKO parasites does not lead to clinical disease. Only WT parasites, but not DKO-parasites, were found in tissues either by histopathologic analysis (as shown in heart sections of STAT-4^null^ animals in panel **c**) or in explantation culture of animals at 6 months post-infection (Table in Supplementary Fig. 9). Hpf, high powered field. This is a compilation of data from 3 separate experiments in the STAT-1^null^ mice and twice in the STAT-4^null^ mice, each with comparable results (5 mice per group in each experiment, *n* = 15 or 10, respectively). *P* < 0.05 in both Kaplan–Meier curves and in parasitemia analysis (2–3 mice per group per experiment, mean values and SD are shown for each timepoint).

Immuno-deficient STAT-1^−/−^ or STAT-4^−/−^ mice, which are hyper-susceptible to *T. cruzi* infection^48,49^ were also inoculated with the DKO strain to test for pathogenicity. As expected, inoculation of either STAT-1^−/−^ or STAT-4^−/−^ mice with WT parasites resulted in high levels of parasitemia and death of 100% of the mice between 15-23 days post-infection (for STAT-1^−/−^) (Fig. 4a) or 21–62 days for STAT-4^−/−^ mice (Fig. 4b). Inoculation with DKO parasites, surprisingly, resulted in no parasitemia, clinical symptoms or death in either immuno-deficient mouse strain. We have repeated these experiments (5 mice per group in each experiment, over 3 experiments in STAT-1^−/−^ nice and 2 in the STAT-4^−/−^ mice) with the same results. We observed the DKO inoculated mice for 6 months (for STAT-4^−/−^ experiments) and 3 months for (STAT-1^−/−^ experiments) without a change in clinical status or survival. Histopathological analysis of the heart tissue of both STAT-1^−/−^ and STAT-4^−/−^ animals (shown for STAT-4^−/−^ mice in Fig. 4c) showed abundant parasite nests in animals inoculated with WT parasites, however, parasites were undetectable in heart tissue of all DKO-inoculated animals. Explantation cultures of tissues (liver, spleen, heart, GI mesentery, stomach, and large intestine) harvested from DKO-inoculated STAT-4^−/−^ mice did not yield parasite growth after 4 weeks of observation whereas parasites were found in the majority of tissue explant cultures (spleen, heart, GI mesentery, stomach and large intestine) of mice inoculated with WT parasites (Supplementary Fig. 9). These results indicate that even in immuno-deficient mice, the DKO parasite line is unable to mount a productive infection. The level of parasitemia in WT-inoculated STAT mutant mouse strains is notably higher than in immuno-competent mice (Fig. 3) probably due to the lack of the ability of the immuno-deficient mice to control parasite growth. Interestingly, there is delayed mortality in the infected STAT-4^−/−^ compared to the STAT-1^−/−^ mice, which we do not completely understand but may be due to differences in the immune defects between the two strains.

### DKO parasites induce anti-*T. cruzi* immunity that protects against acute Chagas disease

**Fig. 5:**
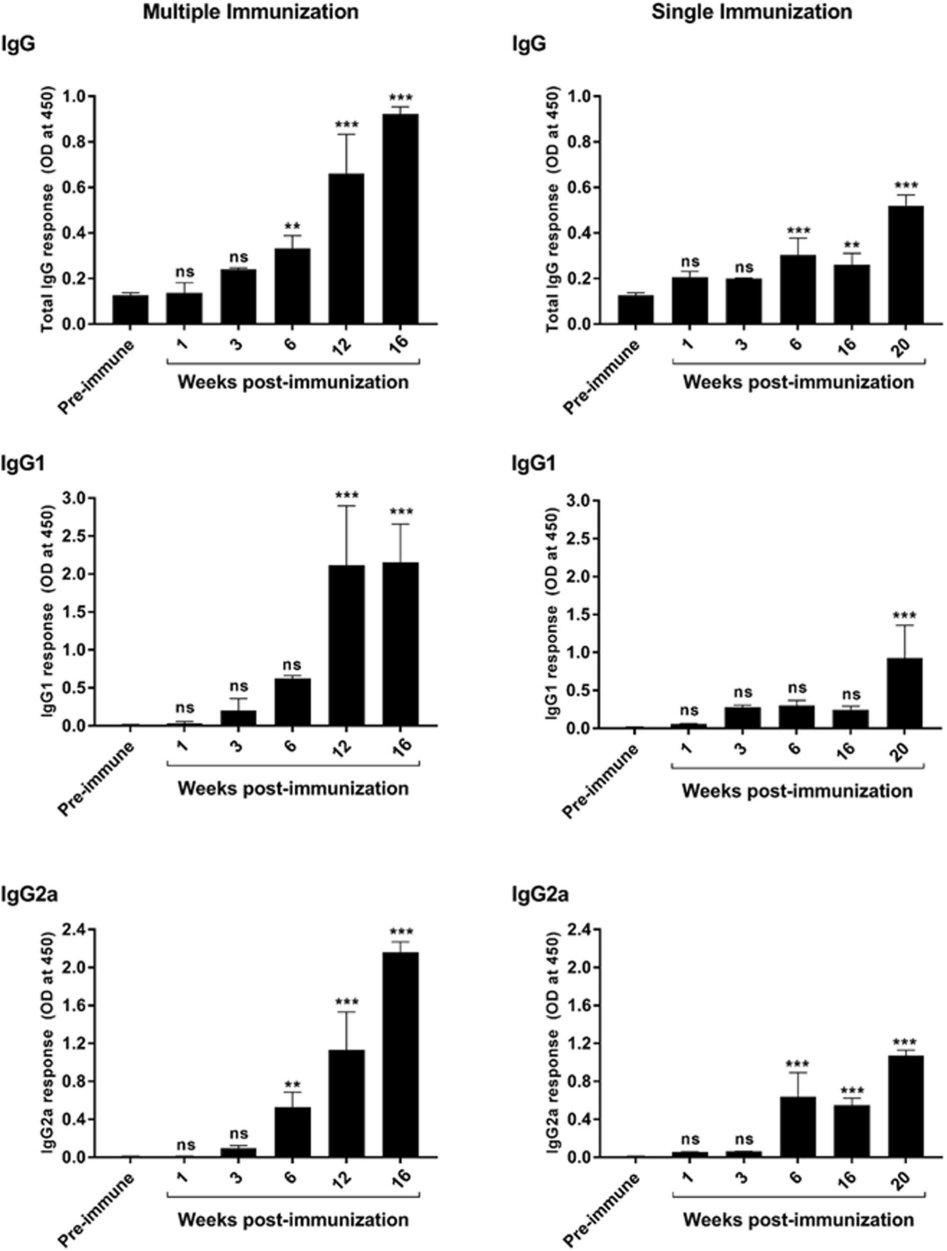
Induction of *T. cruzi*-specific B-cell responses by DKO parasites. Anti-*T. cruzi* total IgG, IgG1, and IgG2a levels after repeated administration of DKO culture-derived metacyclic trypomastigotes in immuno-competent mice (left panels) and also in animals which underwent single DKO inoculation (right panels). Total WT parasite lysates were used as antigen to test serum antibodies using ELISA. Sera were compared using 1:100 dilution. *N* = 5 for each analysis and the mean values and SD are shown for each. One-way ANOVA using Dunnett’s post test was used and *P* values are: **<0.01, ***<0.001.

**Fig. 6:**
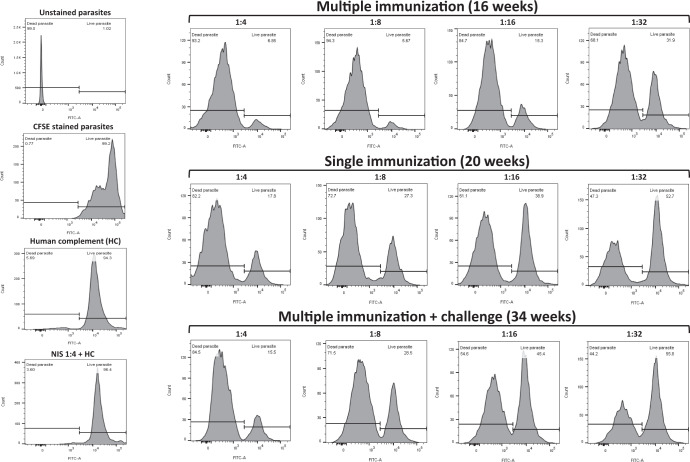
Trypanolytic activity of immune serum from DKO-inoculated animals. Serum collected from animals which were inoculated multiple times (at 16 weeks from first inoculation, upper right panel), after a single inoculation (at 20 weeks post inoculation, middle right panel) and after multiple immunizations WT + challenge (34 weeks after challenge, bottom right panel). WT RHM-derived trypomastigotes were used to analyze for the presence of Ab-mediated complement fixing activity. Both serum from multiple and single administrations of DKO parasites were able to induce trypanolytic activity. Dilution of the serum resulted in incrementally less trypanolytic activity as expected. Control reactions (left panels) included non-stained parasites, CFSE-stained parasites alone, stained parasites incubated in human complement alone and those incubated in non-immune control serum + human complement. Sera were pooled from 3–4 mice per group prior to analysis. See Supplementary Fig. 11 for gating strategy for flow cytometry.

**Fig. 7:**
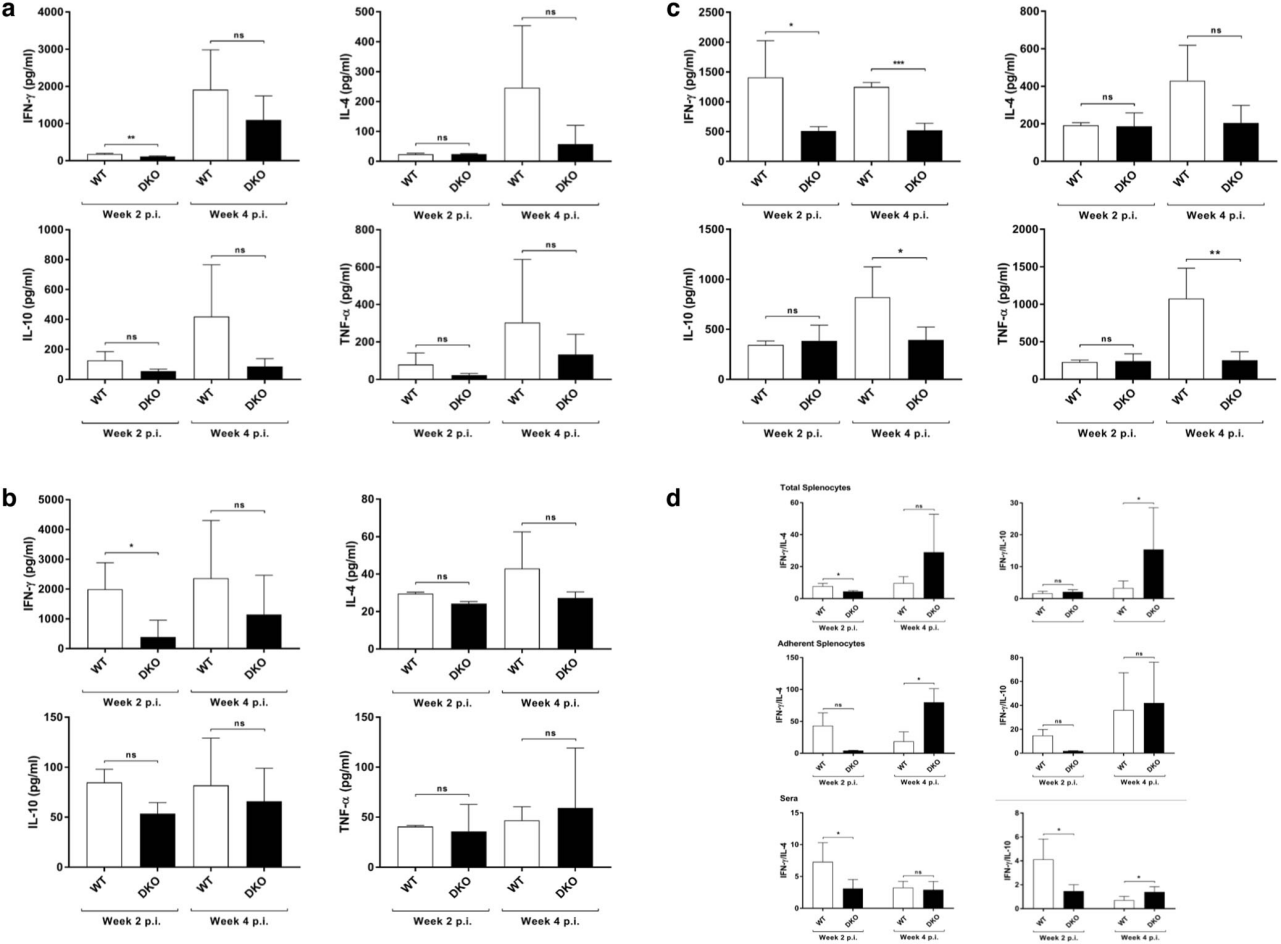
Cytokine responses of mice inoculated with WT and DKO-parasites. Cytokine analysis from total splenic cells (**a**), adherent splenocytes (**b**) harvested from WT and DKO-inoculated animals (at 2 weeks and 4 weeks post inoculation) stimulated with *T. cruzi* antigen. Panel **c** shows cytokine levels of sera of the same animals. Panel **d** shows the ratio of pro- (IFN-γ) and anti-inflammatory (IL-4 or IL-10) cytokines from total- and adherent-splenocytes and sera of inoculated animals. *N* = 4 for each analysis and the mean values and SD are shown for each. For panels **a**–**c**, Student’s t test was used and for panel **d**, Kruskal–Walis test was used. *P* values are: ns, no statistical difference; *<0.05 and **<0.01.

**Fig. 8:**
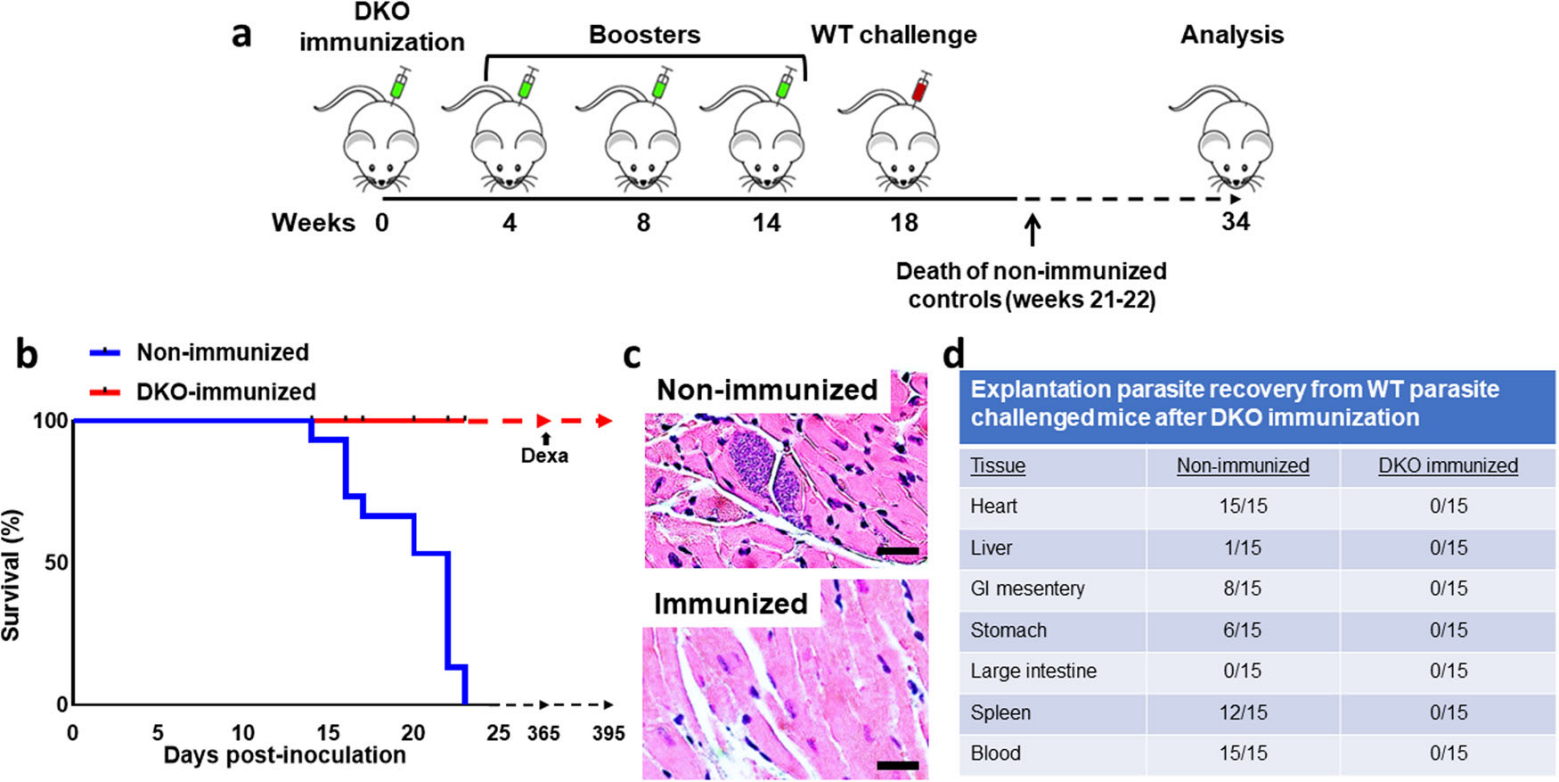
Challenge of mice immunized with DKO parasites. **a** Timeline of immunization and infection of A/J mice. Mice were immunized with 10^5^ cell-culture-derived metacyclic trypomastigotes and then given three booster doses at indicated times with the same amount of metacyclics followed by challenge with WT RHM-derived trypomastigotes 18 weeks after the first dose of DKO parasites. Non-immunized mice served as controls. Terminal analyses of DKO-immunized mice were conducted 16 weeks after WT challenge. **b** Survival curve of WT-challenged non-immunized control mice and DKO-immunized mice. DKO-immunized mice survived WT challenge whereas all non-immunized mice died within 14–23 days post-challenge. In replicate experiments, WT-challenged DKO-immunized animals survived up to one-year post-challenge. Treatment of these animals with dexamethasone (Dexa) does not induce clinical disease, alter survival or cause the emergence of parasites from tissues of any animal (Supplementary Fig. 13). **c** Histopathologic analysis of heart sections of WT-challenged non-immunized (upper panel) and DKO-immunized mice (lower panel). **d** Explantation of indicated tissues in parasite culture medium from WT-challenged non-immunized or DKO-immunized mice 6 months (for DKO-immunized animals) post-infection. This is a compilation of data from 3 separate experiments with comparable results (5 mice per group in each experiment, *n* = 15). The Log-rank (Mantel-Cox test) was used for the Kaplan–Meier analysis. *P* < 0.05.

Because DKO parasites do not appear to successfully replicate in or develop TCTs in vitro or in mice, we tested the ability of repeated inoculations of DKO parasites to produce anti-*T. cruzi* immune responses. Repeated IP inoculation of immuno-competent AJ mice with 10^5^ DKO parasites produced increasing levels of parasite-specific IgG, indicating the development of an anti-parasite B-cell response which included rising levels of IgG subsets of IgG1 and IgG2a (Fig. 5 and Supplementary Fig. 10). This anti-parasite antibody was tested for its ability to induce complement-mediated lysis of trypomastigotes. Flow cytometry-based analysis of immune serum from DKO-inoculated animals demonstrated potent trypanolytic activity (Fig. 6 and Supplementary Fig. 11), whereas control reactions with non-immune serum and complement, or complement alone had no anti-parasite effect. Both non-adherent and adherent splenic cells and sera harvested from WT inoculated animals (n = 4) produced increased levels of IFN-γ at 2 weeks post infection that was significantly higher than that found in DKO-inoculated animals (Fig. 7a–c). At 4 weeks post infection both groups produced increased levels of IFN-γ, IL-4, IL-10, and TNF-α from splenic cells that were not statistically different between the groups, however, the trend was for higher mean levels in the WT-inoculated animals. This is likely due to the increasing number of parasites in the WT inoculated group. Systemic levels of IFN-γ, IL-10 and TNF-α were higher in the sera of WT-inoculated animals (Fig. 7c). Interestingly, the ratios of IFN-γ/IL-4 and IFN-γ/IL-10 were elevated in splenocytes and the IFN-γ/IL-10 was elevated in sera of DKO-inoculated animals at later stages of infection indicating they have pro-inflammatory reactivity to parasite antigen (Fig. 7d). Analysis of IL-17 and IL-13 showed no significant differences between WT- and DKO-inoculated animals (Supplementary Fig. 12). We tested whether the anti-parasite immunity developed by repeated DKO injections was able to protect mice from development of acute disease or death from challenge with WT parasites (Fig. 8). Inoculation of non-immunized mice with WT parasites, as expected, resulted in the onset of clinical infection and death between 2–3 weeks post-infection of all mice (Fig. 8a, b). Wild-type challenge of mice previously “immunized” with repeated injections of DKO parasites did not result in clinical disease and all mice remained completely healthy up to 16 weeks post-challenge (Fig. 8b). We found no evidence of viable parasites in the tissues of DKO immunized WT-challenged animals using explantation analysis (Fig. 8d). We have repeated this multi-inoculation immunization experiment 3 times with the comparable results (with a total number of 15 animals per group). PCR analysis of cardiac tissue of animals 16 weeks post-challenge indicated the presence of parasite DNA using *T. cruzi*-specific primers (Supplementary Fig. 7) indicating deposition of parasites in tissue. This was likely from WT parasites during challenge, since we were no longer able to find NEO-specific amplification suggesting a loss of parasite DNA in tissues from the DKO immunizing strain. In order to test for potential outgrowth of latent parasites, DKO-immunized WT-challenged animals were treated with dexamethasone approximately one-year post-challenge. After 30 days of dexamethasone treatment, we observed no clinical infection in any animal (5 in the group) (Fig. 8b) or the presence of parasites in explantation organ culture or histopathologic analyses of cardiac tissue (Supplementary Fig. 13).

### A single immunization with DKO parasites is sufficient for protective immunity

**Fig. 9:**
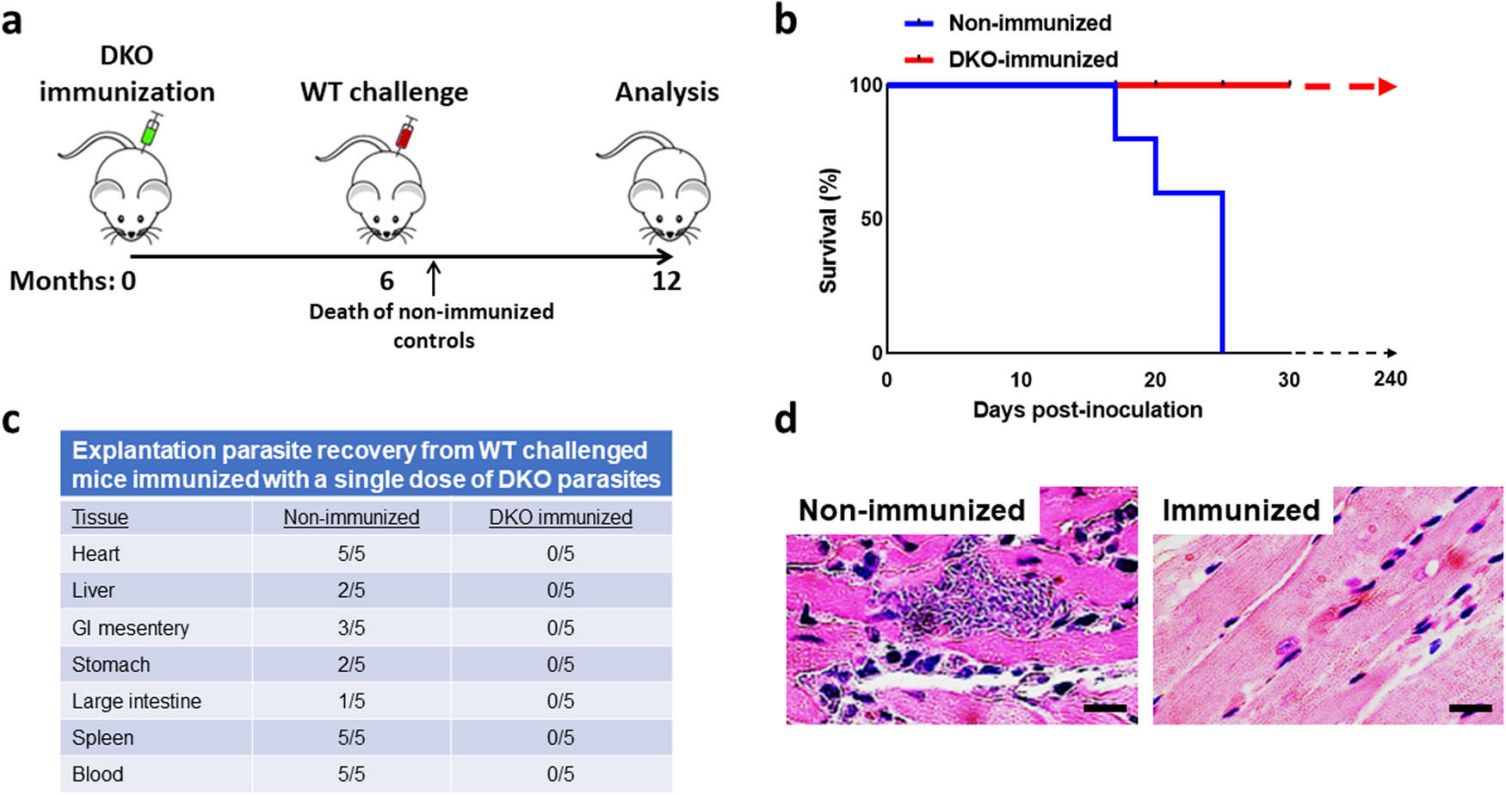
Single dose DKO immunization of mice is protective against lethal challenge. **a** Timeline of immunization and WT challenge of mice. Mice were given a single DKO immunization with 10^5^ parasites then challenged with WT parasites 24 weeks post-immunization (*n* = 5). Non-immunized mice served as controls. Terminal analysis of DKO-immunized mice was conducted 24 weeks after WT challenge. **b** Survival curve of WT-challenged non-immunized control mice and DKO-immunized mice. All DKO-immunized mice survived WT challenge whereas all non-immunized mice died within 18–25 days post-challenge. Challenged DKO-immunized animals were observed for up to 240 days post challenge and no animals developed clinical disease. Treatment of these animals with dexamethasone (Dexa) does not induce clinical disease, alter survival or cause the emergence of parasites from tissues in any animal in the group (not shown). **c** Explantation of indicated tissues in parasite culture medium from WT-challenged non-immunized (at or near the time of death) or DKO-immunized mice 240 days post-infection. **d** Histopathologic analysis of heart sections of WT-challenge non-immunized (left panel) and DKO-immunized mice (right panel). This experiment contained 5 mice per group. The Log-rank (Mantel-Cox test) was used for the Kaplan–Meier analysis. *P* < 0.05.

Our experiments up to this point indicated that four doses of DKO parasites given over a span of 4.5 months induced effective protective immunity to acute disease from WT challenge. We next tested whether a single dose of DKO parasites could elicit protection (Fig. 9). DKO parasites were administered in a single inoculation. Analysis of serum samples of these mice over 20 weeks post-inoculation produced increased titers of anti-parasite total IgG and IgG1 and IgG2a (Fig. 5a, right-sided panels). These mice were then challenged with WT parasites 6 months after immunization. Observation of immunized animals over 6 months post-challenge resulted in no evidence of clinical infection in any animal (Fig. 9b) whereas, as expected, 100% of non-immunized animals died within 21–25 days post-challenge (Fig. 9a, b). Explantation culture and histopathologic analyses indicated the presence of parasites only in the non-immunized challenged animals (Fig. 9c, d).

## Discussion

This report describes the attenuation of *Trypanosoma cruzi* by removal of two of three genes encoding cyclophilin 19. The double-knock-out (DKO) mutant parasite line with depleted Cyp19 expression is viable in culture, but has a diminished growth rate, and is defective in its ability to reach the peak density found with wild-type parasites, suggesting a defect in replication as epimastigotes. The mutant line differentiates rapidly into metacyclic trypomastigotes, further suggesting a defect in normal cell cycle control. We surmise that the rapid conversion of epimastigotes to metacyclic forms at early points in cultivation of cells contributes to the inability of these cells to reach higher densities. The replication of low numbers of epimastigotes prior to their differentiation into metacyclic forms (which are non-replicative) is the key to their continued survival in culture. The DKO metacyclics retain their resistance to complement-mediated killing by normal human serum and are morphologically similar to those of wild-type parasites, suggesting that depletion of Cyp19 expression does not affect these properties. When used to infect mammalian host cells, metacyclic parasites enter cells and form scant amastigotes-like bodies however they do not replicate and fail to give rise to cell-derived trypomastigotes (TCTs). Prolonged observation of DKO inoculated cultures, up to several weeks, fail to show replication of additional amastigotes or the production of TCTs, indicating a profound defect in their ability to replicate intracellularly, differentiate into TCTs and exit host cells. Since Cyp19 is likely involved in the maturation and expression of other parasite proteins, its absence likely affects a multitude of pathways, including both constitutive house-keeping proteins (e.g., cell cycle machinery, differentiation, etc.) as well as those involved in intracellular replication. Thus, the DKO line probably has multiple defects in several hitherto unknown pathways. In light of the finding that released Cyp19 from intracellular amastigotes is important for ROS production, which is critical for replication^45^, the depletion of Cyp19 production in the DKO likely contributes to the inability of parasites to replicate in host cells.

Comparison of the whole genome sequences of single- and double-knockouts with wild-type lines indicate the presence of pre-existing trisomy in chromosome 1 in our Brazil strain, which contains the genes for cyclophilin 19. This was unexpected since the published genomes of *T. cruzi* indicated that this gene is present as single copy on two alleles. We recloned the initial DKO population, after over one year in culture, and analyzed these by whole genome sequencing. Interestingly, we found trisomy in other chromosomes in the DKO (chr 17 and 22) (Supplementary Fig. 3) each of which contains cyclophilin-like genes. In the absence of reversion of attenuation, it is possible that increases in the number of these genes could compensate for loss of cyclophilin 19 genes for other functions unrelated to virulence. Whether there are defects (small deletions, SNPs, etc.) in the DKO clones, other than diminished Cyp19 expression, to account for their profound attenuation is unknown. Since the completion of these immunization studies, we have successfully inactivated the remaining cyclophilin 19 gene, as well as the NEO and HYG selectable markers, present in the DKO strain, using CRISPR/Cas9 technology. These modified parasites have the same in vitro growth characteristics as epimastigotes of the parental DKO line, and metacyclics of this modified DKO line are unable to produce replicating amastigotes or tissue culture trypomastigotes. This is significant inasmuch as the potential repair of the deleted Cyp19 genes or a duplication event leading to restoration of Cyp19 expression is no longer possible in the revised DKO line strain, eliminating the possibility of loss of attenuation conferred by restoration of full Cyp19 expression. We are currently testing these new lines for infectivity and ability to elicit protection in mice.

Infection of mice with DKO parasites does not lead to the development of clinical disease or death for up to one year of observation. Analysis of tissues from DKO-inoculated animals shows no detectable parasites using histopathology and we are unable to recover parasites from explanted tissues, including those from GI mesentery, heart, spleen, or liver, indicating the absence of viable replicating parasites in the tissue of DKO-inoculated animals. However, parasite DNA was detected by parasite-specific PCR analysis in cardiac tissue of DKO-infected animals after 15 weeks post-infection indicating that parasites or parasite DNA deposit in and remain in tissue. RT-PCR analysis for mRNA expression indicated that there is no detectable expression of parasite mRNA in these tissues. The prolonged persistence of these parasites in tissue in the absence of replication may be important to the development and maintenance of protective immunity or may potentially contribute to eventual tissue damage and development of chronic disease. It may be that successful explantation of the DKO parasites from organs is limited due to the low amount parasites present. Alternatively, these “latent” parasites may not be competent to differentiate into and growth as epimastigotes in culture. Utilization of a bioluminescent-fluorescent-based parasite detection system^50^ with our model may help to resolve some of these questions.

Fortuitously repeated inoculation of mice with the DKO line leads to increasing levels of anti-*T. cruzi* B-cell and proinflammatory cytokine responses. Since B cell responses and production of trypanolytic antibodies are thought to be particularly important in immunity in Chagas^51^, the ability of DKO-immunization to elicit anti-parasite trypanolytic antibodies is particularly significant. This is likely the primary mechanism of protection afforded by the mutant strain. Splenocytes harvested from DKO-inoculated animals produce IFN-γ and lower levels of IL-4 and IL-10 indicative of a Th-1 response, which has also been reported to advantageous to the clearance of parasites^15,16^. Increases in Th1- and Th2-associated cytokines from splenocytes in response to *T. cruzi* antigen suggest that T cells are likely source of these cytokines. DKO-immunized mice challenged with high-dose virulent wild-type Brazil strain *T. cruzi* parasites failed to develop clinical signs of infection and had 100% survival, whereas non-immunized control animals had 100% mortality post-infection. This indicates that the anti-*T. cruzi* immunity elicited by the DKO-parasite immunization was 100% effective at protecting animals from acute Chagas disease. Surprisingly, a single immunization with the DKO line was also effective at providing protection to wild-type parasite challenge 6 months after inoculation. Despite the less robust induction of antibodies than in the multiple immunized animals, the immune response elicited by a single dose was immunogenic and long-lasting. Additional studies will be needed to understand whether the duration of protection is longer and whether the mutant line is protective against development of chronic disease. A potential advantage of the DKO mutant is that it provides exposure of the immune system to a large array of metacyclic stage antigens, which may give rise to a more robust immune response than would subunit vaccines composed of a single or limited number of antigens. In addition, using the whole parasite might serve as a natural adjuvant, bolstering the immune response. We have not found any deleterious effects from administration of the DKO line to animals, regardless this is a potential concern since the development of host immunity to certain parasite antigens can serve to generate autoimmunity through molecular mimicry leading to tissue inflammation^52^. The inability of the DKO to have more robust growth in STAT-1 and STAT-4 deficient mouse strains than in immuno-competent mice suggests that the lack of growth of mutant parasites in tissues of immuno-competent mice may not be related to anti-parasitic immune mechanisms. Induction of immunosuppression using dexamethasone, in immuno-competent mice inoculated with DKO parasites also did not result in recrudesced infection. This further suggests that the outgrowth of the DKO strain, if persistent in tissues at low levels is not amenable to lifting of immune-surveillance. Interestingly, dexamethasone treatment of DKO immunized, WT-challenged animals also did not result in emergence of latent infection of WT parasites, suggesting that they either are not present in high numbers or that they may be unable to remain viable in tissues.

Our results demonstrate that the parasite mutant described here is highly effective in prevention of acute Chagas disease in the mouse model. It is effective in a single dose and appears safe for use on immuno-deficient hosts. Other potential roles of this strain including using this as a heterologous antigen delivery system and immunotherapy in Chagas disease. Further studies are needed to understand if this mutant has the ability to induce cross-protection against infection by other *T. cruzi* strains, determination of the length of protective immunity afforded by a single dose, dose-response analysis, comparison of most effective route of delivery as well as more robust safety testing in other immuno-deficient mouse strains and infection studies in human cell lines.

## Methods

### Parasites and cells

*T. cruzi* Brazil strain epimastigotes were cultured at 26 °C in liver digest-neutralized tryptone (LDNT) medium supplemented with 10% fetal bovine serum (FBS), 20 μg/ml hemin, 100 μg/ml streptomycin, and 100 U/ml penicillin. Early to mid-log-phase parasites were used for the experiments. Transformed clones of G418 and Hygromycin-resistant cells were maintained at a concentration of 200 μg/ml of G418 and Hygromycin B in LDNT medium. Rat Heart Myoblast (H9C2) cells were maintained in Dulbecco’s modified Eagle medium (DMEM) with 10% FBS, 100 μg/ml streptomycin, and 100 U/ml penicillin at 37 °C in a humidified atmosphere containing 5% CO_2_. For challenge experiments, tissue culture trypomastigotes of the wild type were recovered from the supernatants of infected monolayers of H9C2 cells, washed thrice in PBS and resuspended at 10^6^ per mL and 100 μL (10^5^ parasites) were administered by IP injection.

### Animals

All animal experimental protocols were approved by the Ethical Committee for Animal Research of the Ohio State University. Mice were maintained under a 12-h light-dark cycle. The animals were maintained according to the rules and regulations of The University Laboratory Animal Resources (ULAR). *T. cruzi* Brazil strain parasites were maintained in AJ mice (Jackson Laboratories, Bar Harbor, ME, USA). Male AJ mice were inoculated with 1 × 10^5^ trypomastigotes, unless otherwise stated, for all the experiments.

### Plasmid constructs preparation

Plasmids used for Cyp19 knockout were prepared as follows: Cyp19 5′-UTR and 3′-UTR fragments were generated by PCR using genomic DNA as template. The PCR primers used to amplify 442 bp of 5′-UTR were (forward, incorporating NdeI the restriction enzyme site) 5′-GTCTACTACTACCATCCCAAGG-3′ and (reverse, adding an XhoI site) 5′-TGTGTGTTGTTAATAAATTAATTC-3′, and PCR primers used to amplify 177 bp of 3′UTR were (forward, adding a SalI site) 5′-ACTGCTCTTCCGCGCAGAGCTTTG-3′ and (reverse, adding a BamHI site) 5′-GTTAAGTCAGATTTTCACACCC-3′. PCR primer pairs (forward, adding an XhoI site) 5′-ATGATTGAACAAGATGGATTGC-3′ and (reverse, with addition of a SalI site) 5′-TCAGAAGAACTCGTCAAGAAGG-3′ were used to amplify 795 bp of neomycin phosphotransferase (Neo) gene, and PCR primer pairs (forward, with an XhoI site) 5′-ATGAAAAAGCCTGAACTCACC-3′ and (reverse, with a SalI site) 5′-CTATTCCTTTGCCCTCGGACG-3′ were used to amplify 1026 bp of hygromycin phosphotransferase (Hyg) gene. The amplified products of the 5′- and 3′-UTRs, Neo and Hyg genes were cloned into pGEM®-T Easy (Promega, Madison, WI, USA) vector. The gene cassettes of 5′-UTR + Neo/Hyg+3′-UTR were constructed in pET15b and purified using plasmid maxi prep kit columns (Qiagen). Modified pET15b plasmid was digested with NdeI and BamHI and the entire assembled cassette fragments were purified and used for parasite transfection.

### Genomic DNA/RNA isolation

Epimastigotes from *T. cruzi* wild type (WT) and Cyp19 knock out (KO) strains were collected from the culture and washed twice with PBS. Genomic total DNA and RNA were extracted using Trizol (Thermo Fisher Scientific), DNeasy Blood & Tissue Kits (Qiagen), and Trizol, respectively, according to the manufacturer’s protocol. PCR analysis was performed to amplify various targets to clone into plasmids and to confirm the presence and absence of genes in knock-out experiments. For PCR and RT-PCR analysis of tissues, primers (TCZ1 and TCZ2) for *T. cruzi*-specific microsatellite sequence were used to determine the presence of parasite DNA and the expression of mRNA by parasites in tissue. For RT-PCR experiments, primers specific for mammalian actin were used as internal control and normalization of values and calculation of −2^ΔΔCT^.

### Transfection, selection, and cloning of *T. cruzi*

A total of 1 × 10^7^ early log phase of *T. cruzi* epimastigotes were used to transfect with 50 μg of linearized Neo/Hyg plasmid using the electroporator (Harvard Apparatus BTX, Holliston, MA). Transfected parasites were maintained for 24 h in LDNT medium alone. The selection pressure was created with 200 μg/ml of G418 and 200 μg/ml of Hygromycin B for transfectants with neomycin phosphotransferase and hygromycin phosphotransferase gene cassettes, respectively. Parasites were selected for 4-5 weeks post-transfection. Individual clones were obtained by limiting dilution into a 96-well plates. Individual cloned cells were further analyzed for the absence of Cyp19 genes.

### Nuclear genomic sequencing and analysis

The libraries of total genomic DNA isolated from the indicated clones were sequenced using a PE75 protocol (75-bp reads from each paired end) on a HiSeq2000 at the UW Sequencing Northwest Genomics Center. After de-indexing, we obtained between 21.2 M (JM255) and 44.1 M (JM256) reads per library. Aligned all reads from the 7 WGS libraries against the 43 chromosomes of the TcBrA4 genome from TriTrypDBv46 using Geneious assembler containing the following loci: (1) Cyp19: A 21,328-bp fragment from PRFA01000019 (reverse complemented) containing three genes on either side of C4B63_19g183), which is the orthologue of TcCLB.506925.300. This locus is on Chr39-S in CL Brener; (2) Cyp11: A 12,966-bp fragment from PRFA01000149 containing 4–5 genes on either side of a paralogue (C4B63_149g20) of TcCLB.506925.300. This locus is on Chr22-S in CL Brener. The alignments were manually examined and the reads-per-kilobase-per-million (RPKM) calculated for each of the contig in order to compare gene copy numbers. Depending on the library ~10–20% of the reads did not align. Chromosomal number was estimated in each sample using two different approaches: (1) Normalization of the mean read coverage for each chromosome by dividing by the median of all 43 chromosomes and multiply by 2; (2) Normalization of the RPKM (from Geneious) for each gene by dividing by the median for all genes and multiplying by 2 to generate the “copy number” of each gene, allowing to calculate the copy number mean for all genes on each chromosome.

### Western blot

Cells were collected and washed twice with PBS and lysed with RIPA lysis buffer (Pierce, Thermo Fisher Scientific, Waltham, MA, USA) containing protease inhibitor cocktail (Roche Diagnostics, Burgess Hill, UK). Total protein concentration was measured with Pierce^TM^ BCA Protein Assay Kit (Thermo Fisher Scientific, Waltham, MA, USA). Total protein lysates were mixed with SDS-PAGE Protein Loading Buffer Blue (National Diagnostics, Atlanta, GA), and boiled for 5 min. Proteins were separated in 15% polyacrylamide gels and transferred to nitrocellulose membranes. The membranes were blocked with 5% skimmed milk containing Tween 20 (0.5%) for 1 h. The membranes were probed with primary antibodies (1:2000 dilution) at 4 °C overnight followed by the incubation with the corresponding secondary antibodies (at 1:2000 dilution) labeled with horseradish-peroxidase (anti-mouse IgG Ab, catalog #7076S, Invitrogen; anti-rabbit IgG, catalog #PAS-28648, Cell Signaling Technology) for 90 min. Polyclonal Cyp19 primary antibodies were prepared in our lab by immunization of mice with recombinant Cyp19. N-terminal specific Cyp19 antibodies were raised in rabbits immunized with a KLH conjugated 10 residue synthetic peptide corresponding of the N-terminus of Cyp19 (New England Peptide, Project #4339). The bound antibodies were detected by enhanced chemiluminescence reagents (Millipore, Burlington, MA, USA). Visualization of the transferred protein was done with FluoroChem HD2 (Protein Simple, CA, USA). Primary antibodies used were either polyclonal anti-Cyp19 antibodies produced in mice using purified his-tagged recombinant Cyp19 purified from *E. coli* or anti-peptide antibodies produced in rabbits using the unique 10 amino acid N-terminal region of Cyp19. Source data for western blotting, PCR analysis of knock-out parasite lines, and for PCR analysis of cardiac tissue are found in Supplementary information.

### Histopathology

Heart tissues from mice were fixed in 10% buffered formalin. Tissue sections (5 μm) were stained with Haematoxylin-Eosin (H&E) and examined under light microscope for parasites. Images were taken using a LEICA DMi1 using the LAS V4.12 software. Up to 20 fields (10–40x) were examined for each animal (*N* = 5 per group over 2–3 replicates in each experiment).

### Explant culture

Explants of organs from *T. cruzi*-inoculated mice were cultured in LDNT medium. Briefly, organs were collected and excised into smaller pieces and put into 25 cm^2^ non-vented flask containing 10 ml LDNT medium. Cultures were incubated at 26 °C and examined twice a week for any growth of parasites for up to 8 weeks.

### Determination of IgG, IgG1, and IgG2a in serum by antibody ELISA and analysis of trypanolytic antibodies

For analysis of IgGs, *T. cruzi* antigen was prepared by lysis of whole cells through rapid freeze-thaw technique. 96-well microplates were coated with *T. cruzi* antigen (5 μg/ml) at 4 °C overnight using antigen coating buffer (phosphate-buffered saline, pH 9.0). Total IgG, IgG1, and IgG2a antibodies against *T. cruzi* were measured from serially diluted serum of un-inoculated and inoculated mice. Comparisons between sera in Fig. 5 were done with at 1:100 dilution. The antibody concentrations were measured at a 450 nm wavelength using SpectraMax microplate reader and data were analyzed by Softmax Pro Software (Molecular Devices LLC, Sunnyvale, CA, USA). The presence of trypanolytic antibodies in serum was assessed using carboxyfluorescein succinimidyl ester (CFSE) labeled parasites in a flow cytometry-based system (FACS Celesta, BD Biosciences, using Flowjo version 10 software). Wild-type RHM-derived trypomastigotes were labeled with PBS containing CFSE for 30 min at RT making them fluorescent and detectable in the FITC channel. Labeled parasites were incubated with serial diluted immune serum (pooled from 3–4 mice) followed by human complement for 4 h. Lysis of parasites was measured by diminished CFSE fluorescence in flow cytometry. Control included parasites incubated in non-immune serum + complement, complement alone and heat-inactivated complement.

### Cytokine analysis

Splenocytes were harvested from wild-type mice inoculated with WT or DKO mutant parasites. The cells were plated at a concentration of 4 × 10^6^ cells/ml in RPMI 1640 medium supplemented with 10% FBS, 100 μg/ml streptomycin, and 100 U/ml penicillin, and 1% HEPES. The non-adherent cells were removed and adherent cells (consist of mostly macrophages) were tested separately from total splenocytes. After stimulation of cells with 20 μg/ml *T. cruzi* freeze-thaw whole-cell antigen for 72 h, supernatants were collected, and the production of cytokines were measured by sandwich enzyme-linked immunosorbent assay (ELISA). Serum from inoculated mice were also tested for cytokine levels. Cytokines were analyzed by using Mouse Duoset ELISA kits (R&D Systems, Minneapolis, MN, USA) as per manufacturer’s instructions. Cytokine concentrations were measured at 450 nm with wavelength correction at 570 nm using SpectraMax microplate reader and data were analyzed by Softmax Pro Software (Molecular Devices LLC, Sunnyvale, CA, USA). The spleens for 4–5 animals per group were assayed.

### Production and complement-selection of metacyclic stage parasites

Normal human serum was purchased from Sigma-Aldrich. Late stationary-growth phase epimastigote cultures of either wild type or mutants, containing a mixture of epimastigotes and metacyclics, were collected and washed twice with phosphate-buffered saline. The cells were mixed with normal human serum at a final 30% concentration and incubated at 37 °C for 30 min with intermittent shaking. This process enriches from complement-resistant metacyclics, since epimastigotes are sensitive to complement-mediated killing. The cells were centrifuged at 400 × *g* for 5 min and kept at room temperature for 60 min permitting metacyclic parasites to emerge from the pellet into the supernatant. The supernatant was collected, washed five times with phosphate-buffered saline and the number of metacyclics were counted using improved Neubauer counting chambers. Metacyclics produced in this manner were used in infection experiments comparing wild type and mutants and used for immunization studies with the DKO mutant.

### Scanning electron microscopy

Cells were fixed and processed for SEM at the Campus Microscopy and Imaging Facility (CMIF), The Ohio State University, OH, USA. SEM images were obtained with a FEI Nova NanoSEM 400 Scanning Electron Microscope equipped with secondary and low-vacuum detector with a field-emission gun (FEG) electron source.

### In vitro infections

All in vitro infections were performed in H9C2 and RAW cells. Host cells were plated at a concentration of 1 × 10^5^ cells/well in 12-well plates containing DMEM medium containing 10% HIFBS. Infection of H9C2 cells was performed using serum selected metacyclic-trypomastigotes and incubated at 37 °C. After 24 h, the cells were washed with PBS in order to remove any extracellular metacyclics. The plates were re-incubated in complete medium at 37 °C 1–2 weeks. The formation of amastigotes and trypomastigotes were observed up to 2–3 weeks under light inverted microscope.

### Animal infections

In order to examine the infectivity of *T. cruzi* wild type (WT) and DKO lines parasites, AJ, STAT1^−/−^(BALB/c), and STAT4^−/−^(BALB/c) mice (Jackson Laboratories, Bar Harbor, ME, USA) were inoculated with 1 × 10^5^ serum-selected metacyclic trypomastigotes in 100 μl PBS through intraperitoneal route. Five mice were used in each group for each experiment. Mice were examined daily for clinical symptoms to determine overall survival. All of the mice were examined for parasitemia by collecting blood from the tail vein twice a week. The statistically significant difference in survival between WT and DKO was calculated by Kaplan–Meier survival analysis.

### Immunization and challenge infection in mice

Mouse immunization was performed with serum-selected Cyp19 knock out metacyclic trypomastigotes in AJ mice. Mice were immunized intraperitoneally four times with 1 × 10^5^ trypomastigotes at 0, 4, 8, and 14 weeks. At 18 week, mice were challenged with 1 × 10^5^ *T. cruzi* wild-type tissue-culture trypomastigotes (TCTs) derived from in vitro culture in RHMs. Non-immunized control AJ mice were inoculated with 1 × 10^5^ *T. cruzi* wild-type TCTs. All of the control mice died after ~3 weeks of post-challenge with WT, whereas DKO-immunized mice survived without development of clinical illness (specifically weight loss, huddling behavior, decreased motility and shaking). All of the immunized and challenged mice were harvested at 34 weeks for further evaluation including explant culture and tissue histopathology.

### Statistical analysis

All statistical analyses were done in GraphPad Prism or Excel software. A student *t* test was used to determine statistical significance of differences among the groups, except for Kaplan–Meier survival curves in which the Log-rank (Mantel-Cox) test was used. ANOVA using Dunnett’s post-test for RT-PCR data and the Kruskal Wallis test was used for cytokine ratio analysis. *P* values of <0.05 was considered significant and indicated with a single asterisk (*), double asterisks (**) indicated *P* < 0.01 and triple asterisks (***) indicate a *P* < 0.001.

### Reporting summary

Further information on research design is available in the Nature Research Reporting Summary linked to this article.

## Supplementary information

Supplementary Material

REPORTING SUMMARY

## Data Availability

The authors confirm that the data supporting the findings of this study are available within the article and its Supplementary materials. Use of the DKO strain is patent protected through the Ohio State Innovation Foundation (patent #11110132).
