# Supplementary material for: Immunization with a Trypanosoma cruzi cyclophilin-19 deletion mutant protects against acute Chagas disease in mice
Source: NPJ Vaccines. 2023 Apr 25;8:63. doi: 10.1038/s41541-023-00647-5 (PMC10130101; doi:10.1038/s41541-023-00647-5)
Supplement: Supplementary file 1 — Supplementary Material [file 41541_2023_647_MOESM1_ESM.pdf]

Copy number of Cyp19 genes in the *T. cruzi* lines used in this study

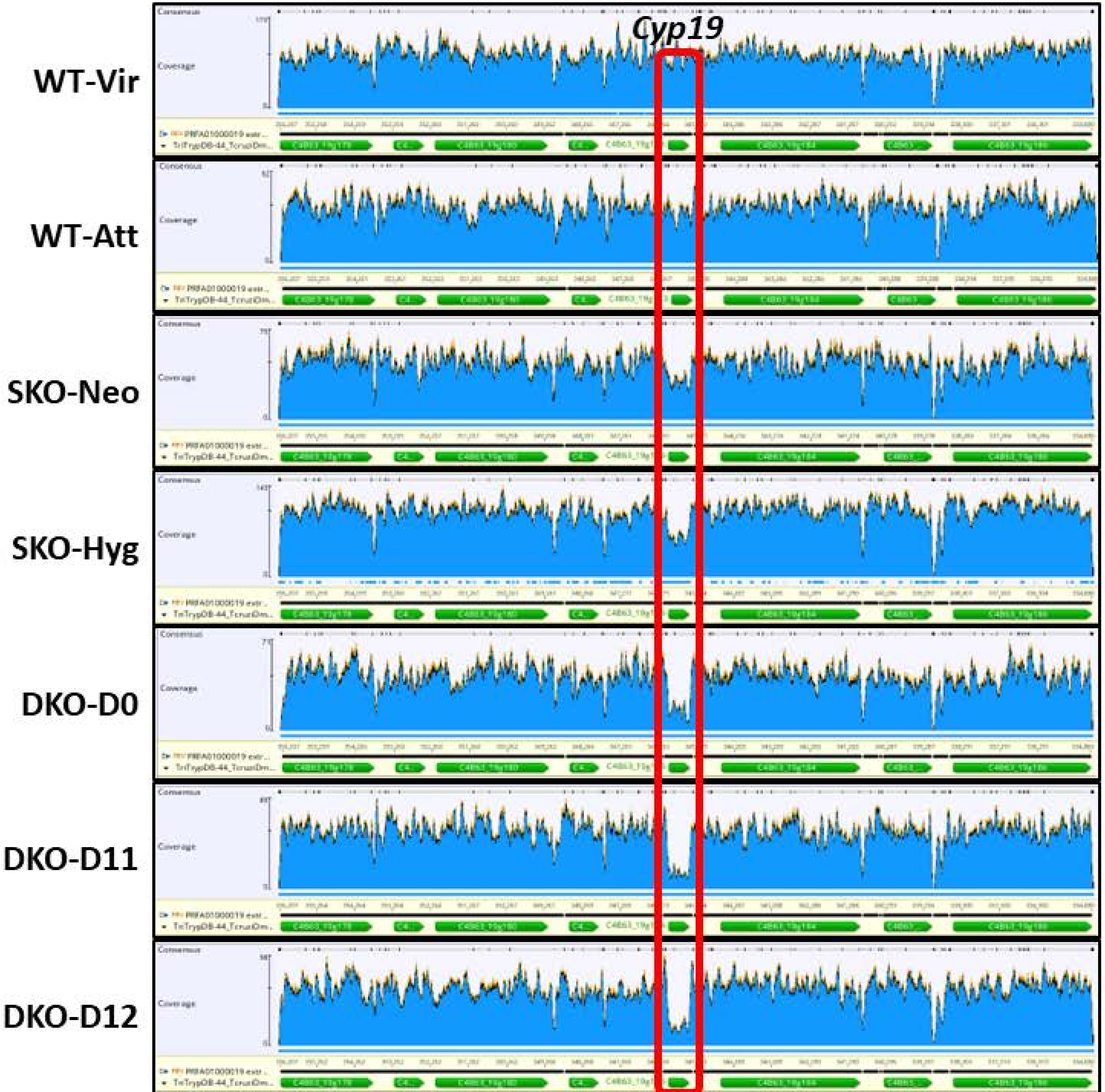

Supplementary Fig. 1: Copy number comparison of the Cyp19 gene copy number in the indicated T. cruzi lines. The relative copy number abundance of Cyp19 (red box) in each of the parasite lines. Single copy knock outs in SKO-Neo and SHO-Hyg show diminished signal relative to WT strains WT-Vir and WT-Att. DKO lines (DKO-D0, DKO-D11 and DKO-D12 show further loss of Cyp19 abundance. The DKO lines show presence of a cryptic 3rd copy of Cyp19.

Somy analysis of *T. cruzi* cell-lines

Mean chromosome coverage

Chr	Length	Wtvir	WTatt	sKOneo	sKOhyg	dKO-D0	dKO-D11	dKO-D12
1	2,738,928	2.1	2.0	2.2	2.2	2.2	2.3	2.2
2	1,986,034	1.8	1.7	1.7	1.8	2.2	2.4	2.3
3	1,768,708	1.7	1.7	1.8	1.8	1.7	1.7	1.7
4	1,676,910	2.1	2.0	2.1	2.2	2.0	2.0	1.9
5	1,492,459	1.7	1.7	1.7	1.8	1.7	1.7	1.7
6	1,421,388	1.6	1.5	1.6	1.6	1.5	1.6	1.5
7	1,369,405	1.9	1.8	1.9	2.0	1.9	1.8	1.8
8	1,336,822	2.0	1.9	2.0	2.0	1.9	1.9	1.9
9	1,155,514	1.6	1.6	1.6	1.6	1.5	1.6	1.5
10	1,097,740	1.9	1.8	1.9	1.9	1.8	1.8	1.8
11	1,076,255	2.1	2.0	2.1	2.1	2.0	2.0	2.0
12	1,041,209	1.9	1.9	1.9	1.4	1.8	1.8	1.8
13	982,025	2.2	2.1	2.2	2.2	2.1	2.2	2.2
14	975,858	2.0	1.9	2.0	2.0	2.2	1.9	2.8
15	969,620	1.9	1.8	1.9	1.9	1.9	1.8	1.8
16	927,191	2.3	2.2	2.2	2.4	2.2	2.3	2.1
17	914,771	2.4	2.3	2.5	2.4	3.5	3.5	3.5
18	909,794	2.1	2.0	2.1	2.1	2.0	2.0	2.0
19	902,532	1.8	1.8	1.8	1.8	1.8	1.8	2.4
20	846,588	1.9	2.1	1.9	1.9	1.9	2.0	2.0
21	820,352	1.4	1.4	1.4	1.4	1.7	1.8	1.8
22	815,970	1.9	1.9	2.0	1.9	2.7	2.9	2.9
23	812,063	2.8	2.7	2.6	2.7	3.3	3.2	3.1
24	778,187	3.1	3.1	3.3	3.2	2.9	3.2	3.1
25	742,617	2.0	2.1	2.0	2.0	1.9	2.0	2.0
26	731,747	1.9	1.9	1.9	1.9	1.9	1.9	1.9
27	716,856	2.1	2.1	2.1	2.1	2.1	2.1	2.1
28	711,759	2.1	2.9	2.5	2.1	2.4	2.4	2.3
29	660,991	1.9	1.8	1.9	1.7	1.8	1.9	1.8
30	660,739	2.0	1.8	1.9	1.5	1.0	0.9	1.0
31	601,716	2.0	2.8	2.9	2.9	2.7	2.8	2.8
32	590,954	1.9	1.9	2.0	2.1	1.9	1.9	1.9
33	574,917	2.1	2.1	2.1	2.0	2.1	2.1	2.1
34	285,003	2.7	2.6	2.6	2.5	2.5	2.6	2.5
35	243,420	1.8	1.8	1.9	1.9	1.8	1.8	1.8
36	231,406	1.3	1.4	1.3	1.4	2.0	2.1	2.1
37	216,495	4.4	4.2	4.4	4.4	3.6	4.1	3.9
38	166,627	1.7	1.7	1.7	1.7	1.6	1.7	1.6
39	160,921	2.3	2.3	2.3	2.3	2.2	2.3	2.3
40	155,078	2.8	3.0	2.7	2.8	2.5	2.7	2.6
41	146,158	2.0	2.0	2.0	1.9	1.9	2.0	1.9
42	141,754	2.8	2.7	2.7	2.8	2.7	2.6	2.6
43	141,550	2.8	2.8	2.9	2.9	2.4	2.7	2.6

Median gene coverage

Chr	WTvir	Wtatt	sKOneo	sKOhyg	dKO-D0	dKO-D11	dKO-D12
1	2.3	2.3	2.3	2.3	2.4	2.4	2.4
2	1.7	1.8	1.6	1.7	2.3	2.5	2.4
3	1.8	1.8	1.8	1.9	1.9	1.8	1.8
4	2.2	2.2	2.2	2.2	2.1	2.1	2.1
5	1.8	1.8	1.8	1.9	1.8	1.8	1.8
6	1.7	1.7	1.7	1.7	1.6	1.7	1.7
7	2.1	2.0	2.0	2.1	2.0	1.9	1.9
8	2.1	2.1	2.1	2.1	2.1	2.1	2.1
9	1.8	1.8	1.8	1.8	1.7	1.8	1.8
10	2.1	2.1	2.1	2.1	2.0	2.0	2.0
11	2.1	2.2	2.1	2.1	2.1	2.1	2.1
12	2.1	2.2	2.1	1.5	2.0	2.1	2.1
13	2.4	2.4	2.4	2.3	2.3	2.4	2.4
14	2.1	2.1	2.1	2.1	2.3	2.1	3.0
15	2.1	2.0	2.1	2.1	2.0	2.0	2.0
16	2.2	2.2	2.1	2.2	2.0	2.1	2.1
17	2.6	2.6	2.8	2.6	3.9	4.0	3.9
18	2.3	2.3	2.3	2.3	2.2	2.2	2.2
19	1.8	1.8	1.8	1.8	1.9	1.8	2.6
20	2.1	2.3	2.0	2.0	2.1	2.1	2.1
21	1.5	1.6	1.5	1.6	1.9	2.0	2.0
22	2.1	2.1	2.1	2.0	2.9	3.1	3.1
23	2.5	2.6	2.5	2.5	2.6	2.6	2.6
24	3.3	3.3	3.3	3.2	2.8	3.1	3.1
25	2.2	2.3	2.1	2.1	2.1	2.1	2.2
26	2.2	2.2	2.2	2.1	2.1	2.2	2.2
27	2.3	2.3	2.2	2.2	2.2	2.3	2.2
28	2.7	3.6	3.0	2.6	2.9	2.9	2.8
29	2.1	2.1	2.1	1.9	2.0	2.0	2.0
30	2.1	2.0	2.0	1.5	1.1	1.0	1.1
31	2.2	3.1	3.1	3.0	2.8	3.1	3.1
32	2.1	2.2	2.1	2.2	2.1	2.1	2.1
33	2.6	2.8	2.6	2.6	2.6	2.8	2.6
34	2.2	2.1	2.1	2.0	2.0	2.1	2.1
35	2.1	2.1	2.0	2.1	2.0	2.0	2.0
36	1.5	1.6	1.5	1.5	2.2	2.3	2.3
37	4.9	4.9	4.8	4.9	4.0	4.6	4.5
38	1.8	1.8	1.8	1.8	1.7	1.8	1.8
39	2.5	2.6	2.5	2.5	2.5	2.6	2.6
40	3.3	3.7	3.2	3.3	2.8	3.2	3.1
41	2.2	2.3	2.2	2.1	2.1	2.2	2.2
42	3.2	3.3	3.2	3.3	3.2	3.2	3.2
43	3.1	3.2	3.1	3.2	2.7	3.0	3.0

Supplementary Fig. 2: Chromosomal number analysis of *T. cruzi* lines used in this study. The overall conclusions of this data:

WT-att and SKOs

- chr37 is pentasomic
- chr24, 28 & 31 are trisomic
- chr1 (central) is trisomic
- chr21 & 36 may be (partially) monosomic
- chr12 & 30 may be monosomic in SKO-hyg

DKOs

- chr2, 17 & 22 become trisomic
- chr1 (left) becomes trisomic
- chr14 & 19 are trisomic in D12
- chr21 & 36 become disomic
- chr30 becomes monsomic

WT-vir

- chr31 is disomic

Partial trisomy of chr 1 from *T. cruzi* strains used in this study

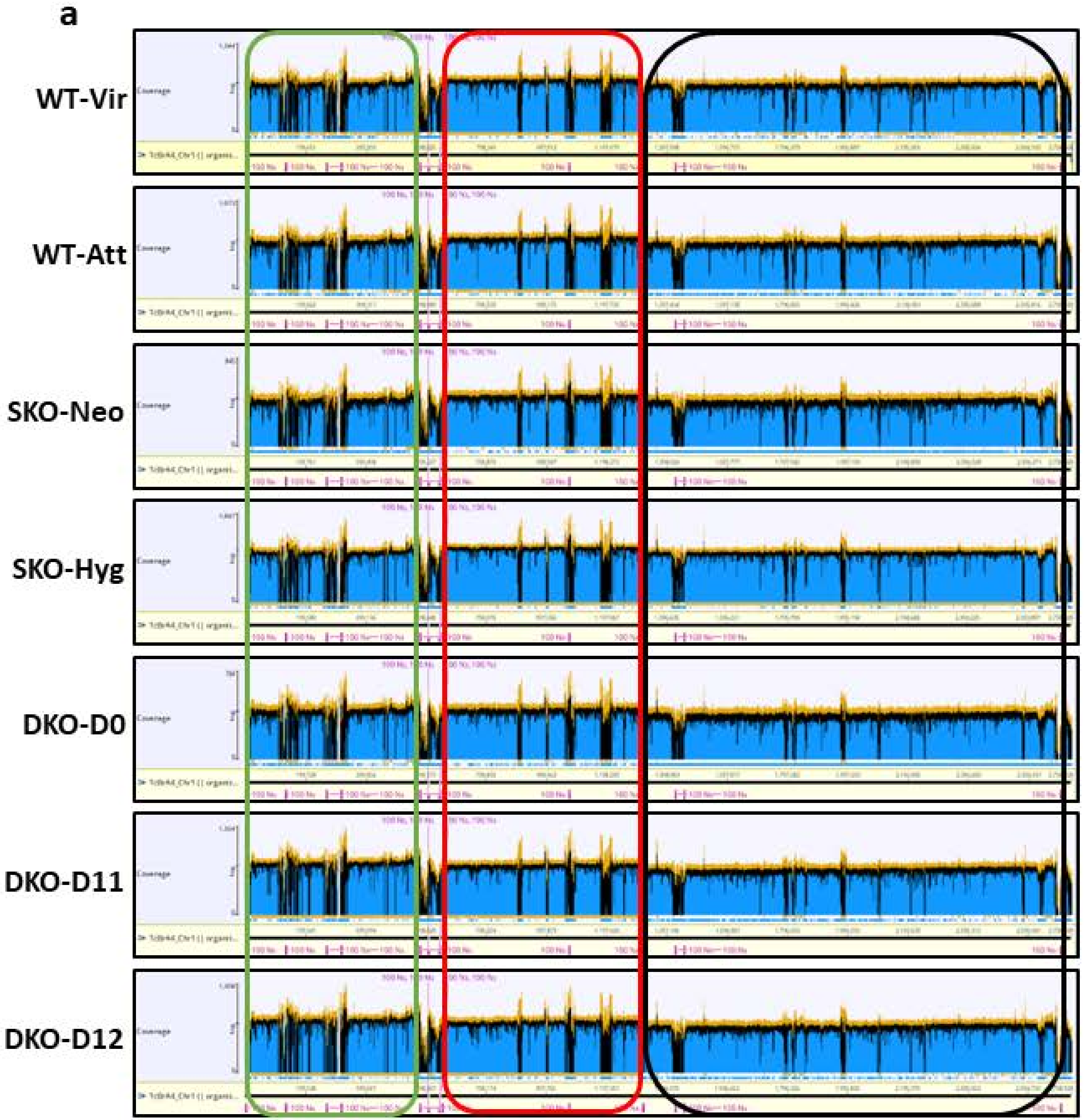

b

Left segment

Chr	Gene ID	Min	Copy number: WTvir	Wtatt	sKOneo	sKOhyg	dKO-D0	dKO-D11	dKO-D12	Gene product
1	TcBrA4_0054880	343094	2.2	2.1	2.0	2.0	2.8	3.6	3.0	hypothetical protein, conserved
1	TcBrA4_0054890	345310	1.9	2.0	2.1	2.0	2.4	3.3	3.0	putative Mg transporter
1	TcBrA4_0054900	347274	2.0	2.0	2.1	1.9	2.6	3.3	3.0	hypothetical protein, conserved
1	TcBrA4_0054910	349729	2.1	2.1	2.2	2.1	2.6	3.3	3.2	hypothetical protein, conserved
1	TcBrA4_0054920	352815	2.0	2.1	2.1	2.0	2.6	3.3	3.0	Protein of unknown function (DUF974), putative
1	TcBrA4_0054930	354874	1.9	2.0	2.0	2.0	2.6	2.9	2.8	WAHD domain of WASH complex, putative
1	TcBrA4_0054940	356930	2.0	2.1	2.1	2.1	2.6	3.7	2.9	60S ribosomal protein L10, putative

Central segment

1	TcBrA4_0056500	840152	2.8	2.5	2.7	2.8	2.8	2.6	2.5	hypothetical protein, conserved
1	TcBrA4_0056510	843656	2.7	2.8	2.7	2.8	2.5	2.8	2.4	mitochondrial ribosomal protein L23
1	TcBrA4_0056520	844651	2.8	2.7	2.9	2.8	2.9	2.7	2.5	hypothetical protein
1	TcBrA4_0056530	846275	2.8	2.4	2.0	1.9	1.1	0.8	1.0	peptidyl-prolyl cis-trans isomerase, putative
1	TcBrA4_0056540	847651	2.7	2.7	2.8	2.9	2.7	2.4	2.7	hypothetical protein, conserved
1	TcBrA4_0056550	851936	2.5	2.7	2.8	3.0	2.5	2.5	2.8	isocitrate dehydrogenase, putative
1	TcBrA4_0056560	853738	2.8	2.7	2.7	2.9	2.6	2.5	2.7	hypothetical protein, conserved

Right segment

1	TcBrA4_0058480	1370743	2.0	1.9	2.0	1.9	2.0	1.8	1.9	hypothetical protein
1	TcBrA4_0058490	1372091	1.8	1.9	1.9	1.9	1.9	1.7	1.8	phosphoglycerate kinase
1	TcBrA4_0058500	1376002	1.9	1.7	1.7	1.9	2.1	1.8	1.8	mRNA export factor MEX67
1	TcBrA4_0058510	1378646	1.9	1.8	1.9	1.9	1.8	1.8	1.9	phenylalanyl tRNA synthetase (beta subunit), putative
1	TcBrA4_0058520	1381075	1.9	1.7	1.9	1.8	2.0	1.8	1.8	hypothetical protein, conserved
1	TcBrA4_0058890	1383870	1.8	2.0	1.8	1.8	1.8	1.7	1.8	hypothetical protein, conserved
1	TcBrA4_0058530	1385824	1.8	1.9	1.9	2.0	1.9	1.7	1.9	hypothetical protein

Supplementary Fig. 3: Partial trisomy of Chr 1: a) representation of gene abundance of Chr 1 in the indicated *T. cruzi* strains. b) comparison of indicated gene copy numbers in different segments of chr 1. The left segment (0-450K) is triploid in the three DKO lines, but diploid in WT and SKO lines. The central segment (660-1320K) is triploid in all lines (this region contains the Cyp19 genes). The right segment (1330-2730K) is diploid in all cell-lines. This suggest that the three segments may be separate chromosomes that fused together or that the large chromosome can fragment during amplification.

Copy number of cyclophilin genes

Chr	Gene ID	Min	Max	Length	Strand	Copy number: WTvir	Wtatt	sKOneo	sKOhyg	dKO-D0	dKO-D11	dKO-D12	Gene product
1	TcBrA4_0056530	846275	846808	534	forward	2.8	2.4	2.0	1.9	1.1	0.8	1.0	peptidyl-prolyl cis-trans isomerase, putative
1	TcBrA4_0060170	1766714	1767778	1065	reverse	1.9	1.8	1.9	1.8	1.8	1.9	1.8	rotamase, putative
1	TcBrA4_0061710	2124841	2125380	540	forward	2.1	2.0	1.8	2.1	2.2	1.9	2.0	rotamase, putative
1	TcBrA4_0063660	2535688	2536821	1134	reverse	2.2	2.2	2.3	2.2	2.2	2.2	2.3	Peptidyl-prolyl cis-trans isomerase C, mitochondrial, putative
3	TcBrA4_0086760	1640620	1641900	1281	reverse	2.1	2.3	2.4	2.1	2.1	2.0	2.1	FK506-binding protein (FKBP)-type peptidyl-prolyl isomerase, putative
6	TcBrA4_0024150	383678	384277	600	forward	2.2	2.2	2.3	2.2	2.0	2.4	2.5	cyclophilin-like protein, putative
7	TcBrA4_0130280	779859	780233	375	forward	2.0	2.1	2.2	2.1	1.9	2.2	2.0	peptidyl-prolyl cis-trans isomerase NIMA-interacting 4, putative
15	TcBrA4_0031220	870048	870848	801	forward	2.0	1.8	2.2	2.3	1.9	2.0	2.2	cyclophilin, putative
16	TcBrA4_0037570	647889	649148	1260	forward	2.3	2.0	2.4	2.1	2.2	2.3	2.2	cyclophilin 15, putative
17	TcBrA4_0138960	746999	747775	777	forward	1.8	2.0	2.4	1.9	3.1	2.8	2.7	rotamase, putative
17	TcBrA4_0139220	834810	835406	597	reverse	9.2	9.0	9.5	9.5	14.6	15.2	15.4	cyclophilin type peptidyl-prolyl cis-trans
18	TcBrA4_0080120	831816	832400	585	forward	1.8	2.0	1.8	2.2	1.8	1.8	1.9	cyclophilin, putative
22	TcBrA4_0009830	377023	377610	588	forward	2.4	2.4	2.6	2.4	3.8	4.0	3.4	cyclophilin, putative
22	TcBrA4_0010680	591462	592181	720	reverse	2.0	1.8	1.9	2.0	2.8	2.5	2.9	cyclophilin-type peptidyl-prolyl cis-trans isomerase, putative
26	TcBrA4_0005970	200282	201250	969	forward	2.0	2.1	2.5	2.1	2.1	2.2	2.1	peptidyl-prolyl cis-trans isomerase, putative
27	TcBrA4_0095700	165435	166361	927	forward	2.3	2.1	2.2	2.2	2.3	2.2	2.3	rotamase, putative
28	TcBrA4_0093420	260516	261781	1266	reverse	2.0	2.9	3.2	1.9	3.1	3.1	3.0	peptidyl-prolyl cis-trans isomerase
28	TcBrA4_0092060	494469	494807	339	reverse	0.4	0.5	0.3	0.3	0.3	0.3	0.3	peptidyl-prolyl cis-trans isomerase, pseudogene
28	TcBrA4_0094370	498046	498744	699	reverse	0.9	1.3	0.7	0.7	0.9	0.7	0.8	rotamase, putative
28	TcBrA4_0094560	548794	549132	339	reverse	2.0	3.3	2.0	2.2	2.0	2.1	2.1	peptidyl-prolyl cis-trans isomerase, putative
28	TcBrA4_0094580	552405	553100	696	reverse	1.6	2.3	1.5	1.5	1.5	1.2	1.3	rotamase, putative
29	TcBrA4_0003380	147771	148100	330	reverse	2.3	2.3	1.9	2.3	1.9	2.5	1.9	peptidyl-prolyl cis-trans isomerase, putative
29	TcBrA4_0003020	540390	541022	633	reverse	2.3	2.8	2.4	2.3	2.5	2.5	2.3	peptidyl-prolyl cis-trans isomerase, putative
31	TcBrA4_0001690	440081	440434	354	forward	2.2	3.4	3.0	3.3	2.7	3.7	3.5	peptidyl-prolyl cis-trans isomerase
41	TcBrA4_0049680	33494	34168	675	reverse	2.1	2.2	1.9	2.1	1.8	2.2	2.0	cyclophilin, putative

Supplementary Fig. 4: Copy number and chromosomal location of cyclophilin genes in the *T. cruzi* lines used in this study. There are 25 genes in the BrazilA4 genome annotated as "cyclophilin", "peptidyl-prolyl cis-trans isomerase" or "rotamase". Cyp19 (TcBrA4_0056530) (has 3 copies in WT, 2 copies in sKO lines and 1 copy in dKO lines. TcBrA4_0138960, TcBrA4_0139220 (chr17), TcBrA4_0009830 and TcBrA4_0010680 (chr22) show increased copy number due to chromosome trisomy in the dKO lines.

Lack of replication of DKO amastigotes

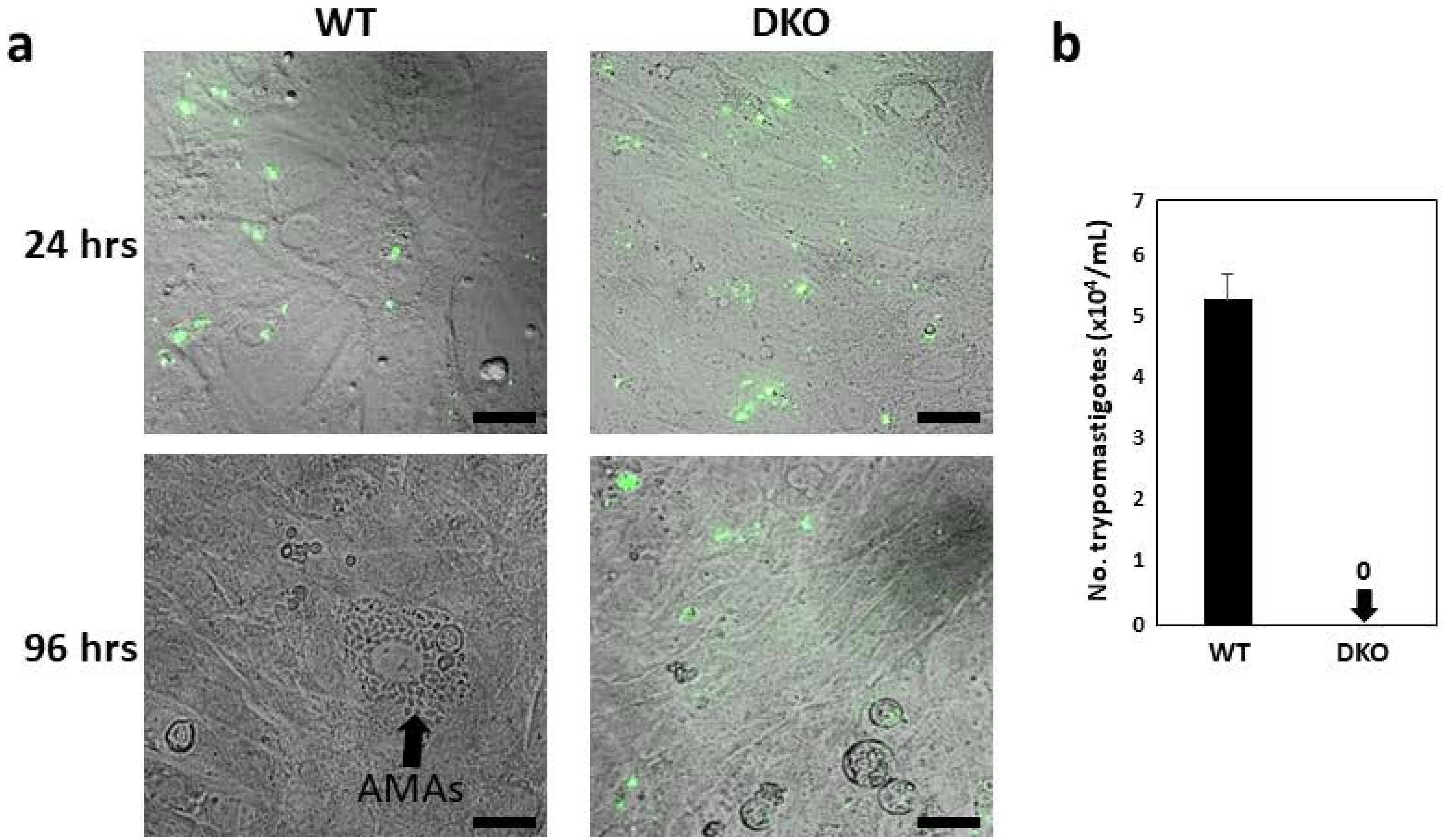

Supplementary Fig. 5: Lack of replication of DKO amastigotes. a) CFSE (carboxyfluorescein succinimidyl ester) was used to label WT and DKO metacyclic parasites which were used to infect RHMs. Cultures were analyzed using fluorescent microscopy up to 96hrs. At 24hrs both parasite lines enter and establish intracellular infection. Thereafter only the WT line replicates developing productive infection diminishing CFSE staining as parasites increase in number, whereas DKO parasites remain quiescent and fail to replicate. b) quantification of medium for production of extracellular trypomastigotes indicates on the WT produces these forms. Mean and SD are show for WT infection. Scale bar = 30μm.

Infection of phagocytic cells with *T. cruzi* cell lines.

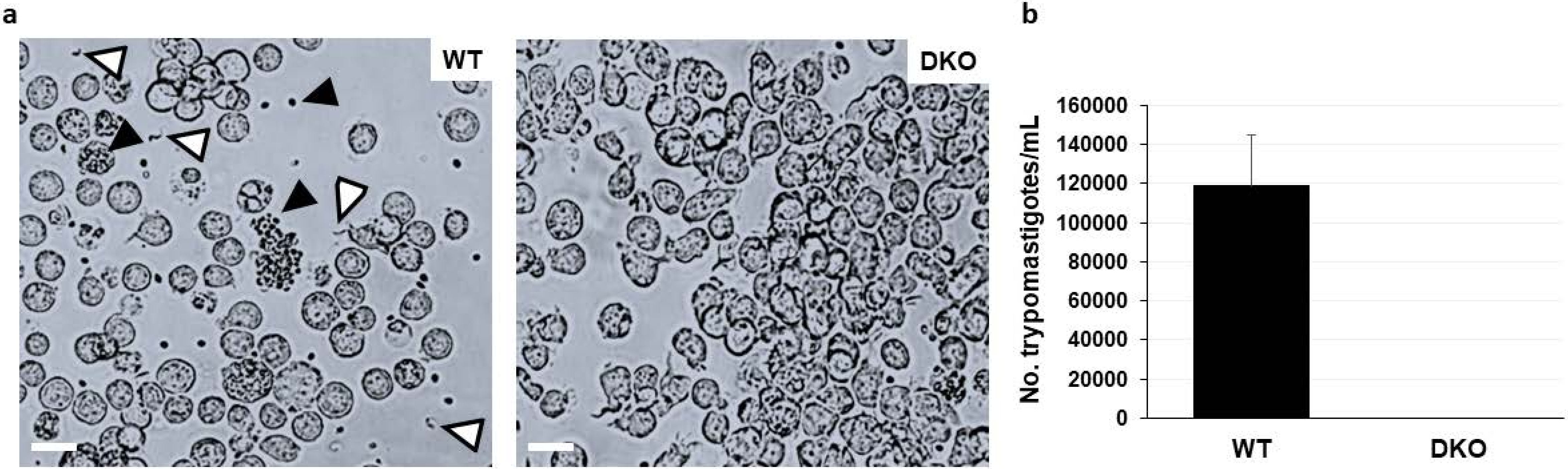

Supplementary Fig. 6: Infection of phagocytic cells with *T. cruzi* cell lines. a. RAW cells were infected with isolated metacyclics of WT and DKO parasites (as indicated) (at a ratio of 10 parasites to one RAW cell) and followed by microscopy for development of infection. WT parasites differentiated into and grew amastigotes (dark arrows) giving rise to extracellular trypomastigotes (white arrows) whereas DKO parasites failed to produce a productive infection and no demonstrable amastigotes or trypomastigotes were observed. b. Quantification of emerging trypomastigotes in the extracellular culture medium at day 6 post infection. Longer incubation of RAW cells infected with DKO parasites did not result in development of trypomastigotes. Mean and SD are shown for WT parasite infection. Scale bar = 25μm.

PCR analysis of cardiac tissue of infected mice

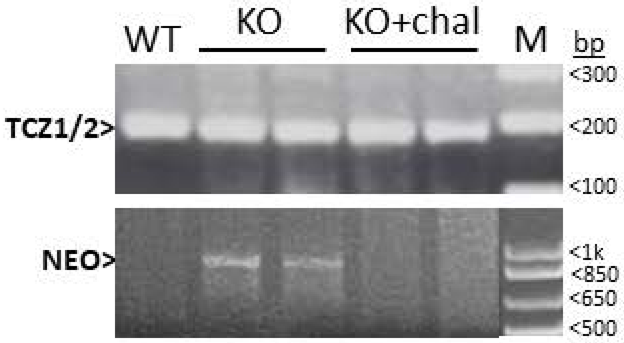

Supplementary Fig. 7: PCR analysis of cardiac tissue of infected mice. Total DNA extracted from formalin-fixed cardiac tissue of AJ mice infected with WT Brazil strain parasites (WT), DKO mutant (KO) or DKO immunized parasites subsequently challenged with WT parasites (KO+chal). Upper panel shows bands for the *T. cruzi* specific TCZ gene using TCZ 1 and 2 primers (182bp). Lower panel shows bands specific for the neomycin phosphotransferase gene (NEO) (855bp). M: 1kb Plus Ladder, Invitrogen. Tissues harvested at: 20 days post infection (WT), 15 weeks post-infection (KO) and 16 weeks post-challenge (KO+chal). See Supplementary Fig. 16 for source data.

RT-PCR analysis of heart and spleen tissue of mice inoculated with WT and DKO *T. cruzi*.

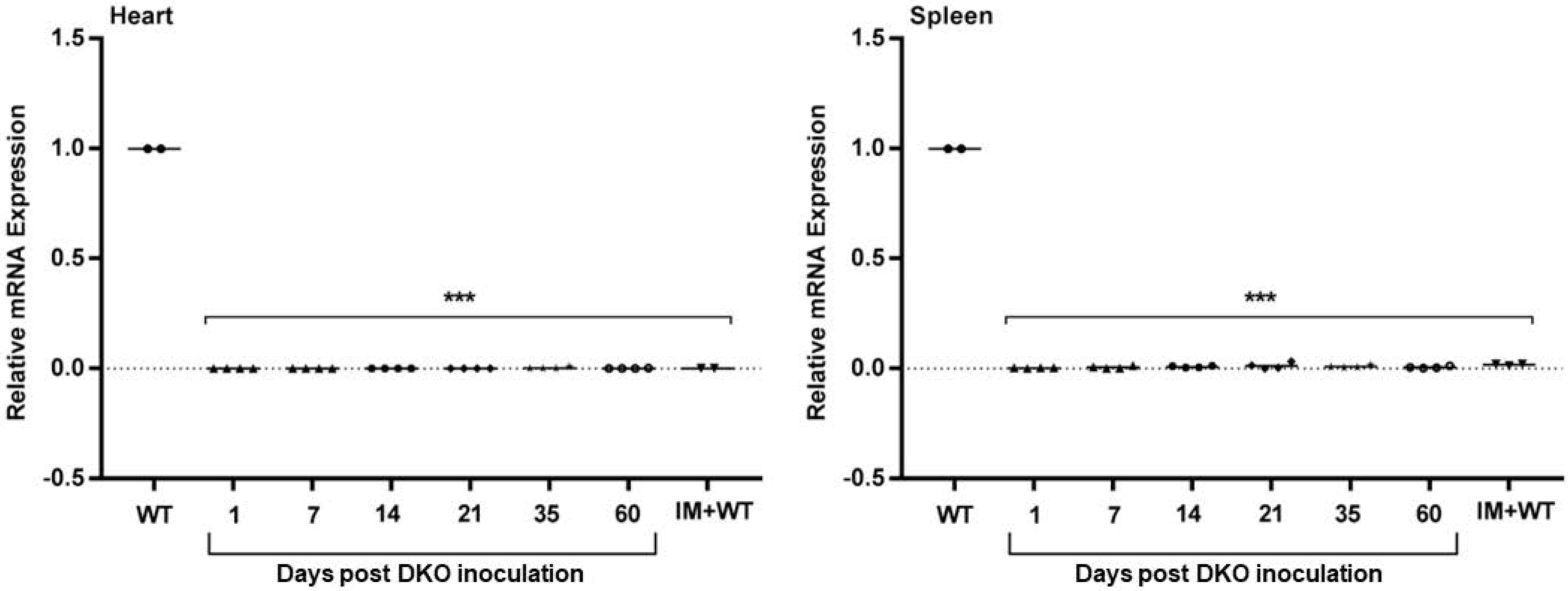

Supplementary Fig. 8: RT-PCR analysis of heart and spleen tissue of mice inoculated with WT and DKO *T. cruzi*. RNA was isolated from heart and spleen tissue of mice inoculated with either WT (positive control), mutant DKO *T. cruzi* and DKO immunized challenged with WT parasites (IM+WT). RT-PCR reactions were amplified using primers for the *T. cruzi*-specific microsatellite region (TCZ). Expression from all samples was normalized with internal control primers for mammalian actin. The data indicates that only animals inoculated with WT parasites alone contain parasites which have active expression from the TCZ region, whereas animals inoculated with DKO parasites, or those immunized with DKO parasites and then challenged with WT parasites have no expression from this region. mRNA expression values are calculated as $2^{-\Delta\Delta Ct}$. The symbols in each category indicated the number of samples tested in each group. Statistical analysis was performed using ANOVA and Dunnett's post-test: ***, P<0.001.

Explantation analysis of organs harvested from STAT-4^{null} mice inoculated with WT and DKO *T. cruzi* lines

Explantation parasite recovery from STAT-4^{null} mice inoculated with WT or DKO parasites		
Tissue	WT	DKO
Heart	10/10	0/10
Liver	3/10	0/10
GI mesentery	5/10	0/10
Stomach	4/10	0/10
Large intestine	4/10	0/10
Spleen	9/10	0/10
Blood	10/10	0/10

Supplementary Fig. 9: Explantation analysis of organs harvested from STAT-4^{null} mice inoculated with WT and DKO *T. cruzi* lines. The indicated tissues were harvested from at the time of death of WT inoculated mice and at 6 months post-inoculation for the DKO-inoculated mice. The numerator refers to the number of animals with out-growth of parasites from the indicated tissue in culture medium and the denominator is the total number of animals analyzed.

Antibody analysis of DKO-immunized mice

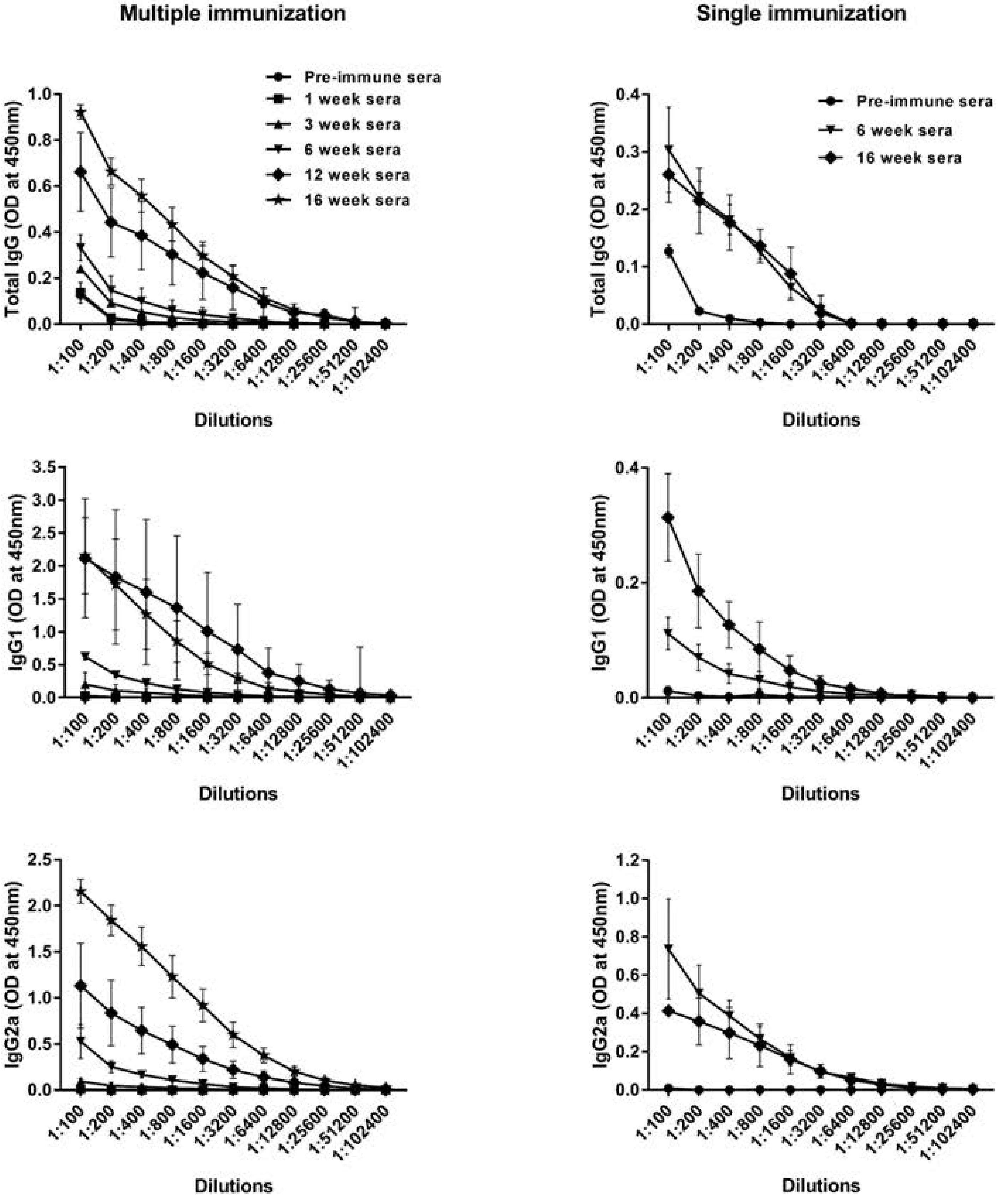

Supplementary Fig. 10: IgG, IgG1, and IgG2a antibody titer of mice. Mice sera collected before and after DKO immunization (sera collected at various time points after multiple and single immunization) was evaluated via ELISA by two-fold serial dilution. Mean values +/- SD from 5 mice per group are shown.

Gating strategy for flow cytometry-based determination of trypanolytic antibody

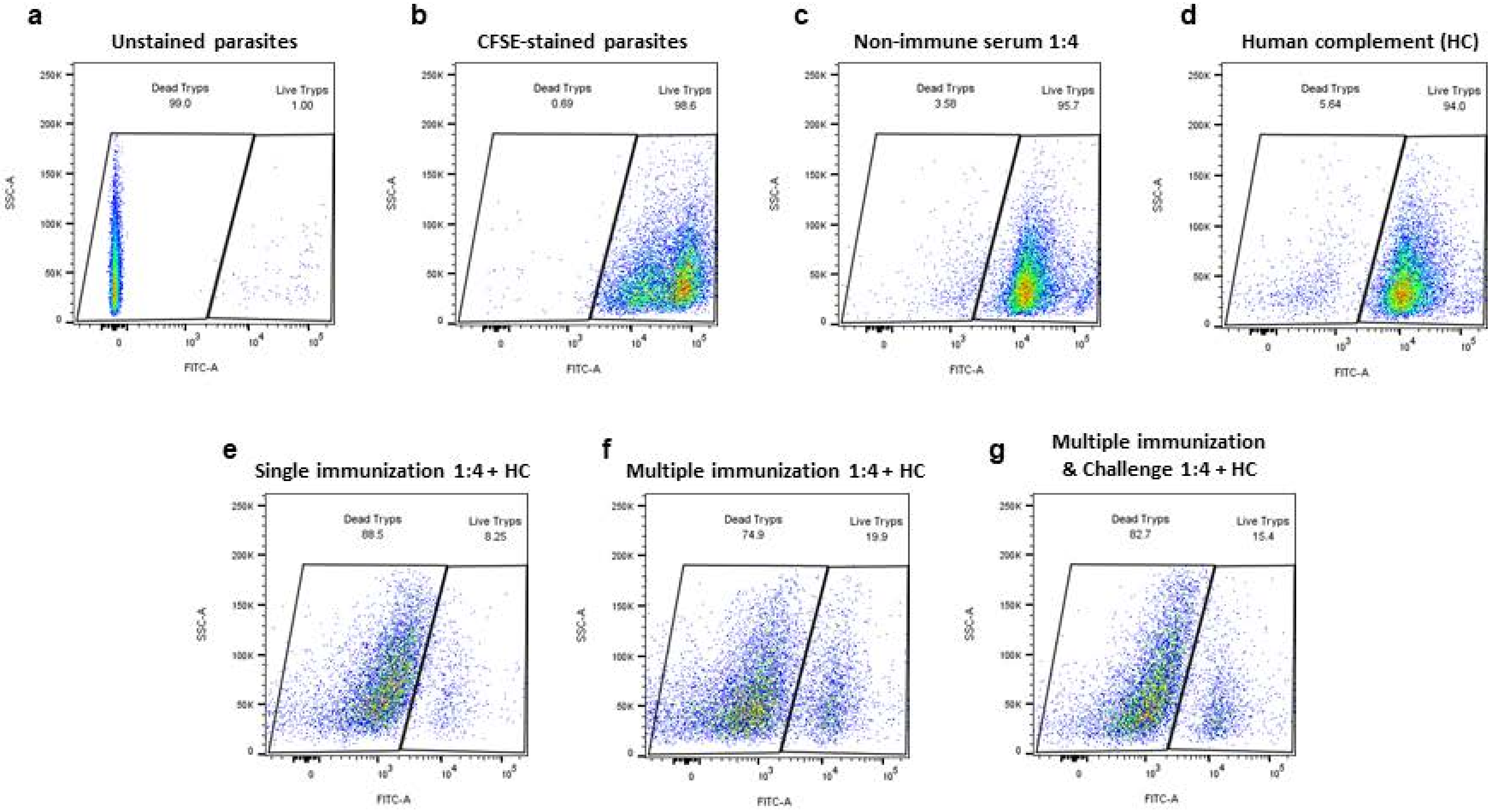

Supplementary Fig. 11: Gating strategy for flow cytometry-based determination of trypanolytic antibody. Panels a & b show unstained and CFSE-stained parasites, respectively. The area of viable parasites ("live tryps") are gated to the right and those with diminished fluorescence ("dead tryps") are gated to the left (as shown). Controls for non-immune serum and complement alone are shown in panels c and d, respectively. Panels e-g show immune serum+HC reactions as shown. Pooled serum from 3-4 animals were used in each assay.

Comparative levels of IL-13 and IL-17 from mice inoculated with WT and DKO parasites

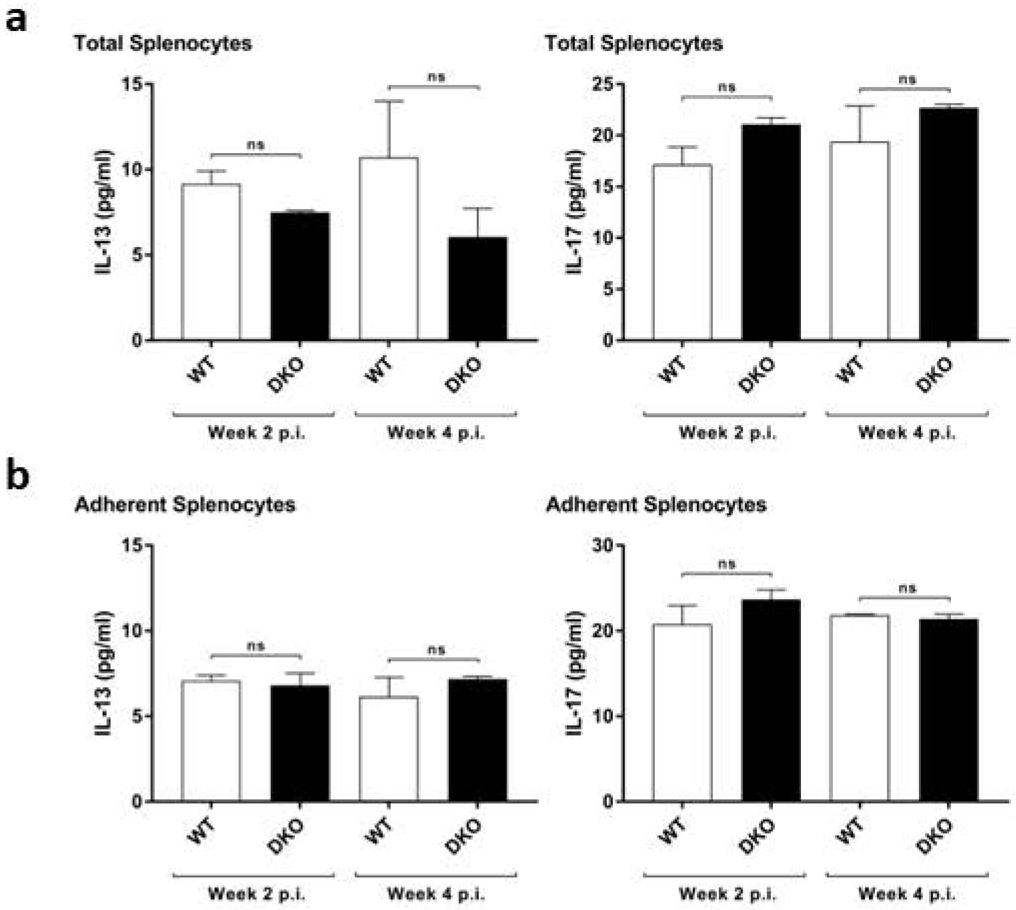

Supplementary Fig. 12: Comparative levels of IL-13 and IL-17 from mice inoculated with WT and DKO parasites. IL-13 and IL-17 levels from total splenic cells (panel a) and adherent splenocytes (panel b) harvested from WT and DKO-inoculated animals (at 2 weeks and 4 weeks post inoculation) stimulated with *T. cruzi* antigen. N=4 for each analysis and the mean values and SD are shown for each. Student's t test was used, and the mean and SD are shown. P values: ns, no statistical difference; * , <0.05 and ** , <0.01.

Analysis of mice inoculated with DKO parasites and treated with dexamethasone

a

Explantation parasite recovery from mice inoculated with DKO parasites and treated with dexamethasone		
Tissue	Non-challenged	WT-challenged
Heart	0/5	0/5
Liver	0/5	0/5
GI mesentery	0/5	0/5
Stomach	0/5	0/5
Large intestine	0/5	0/5
Spleen	0/5	0/5
Blood	0/5	0/5

b

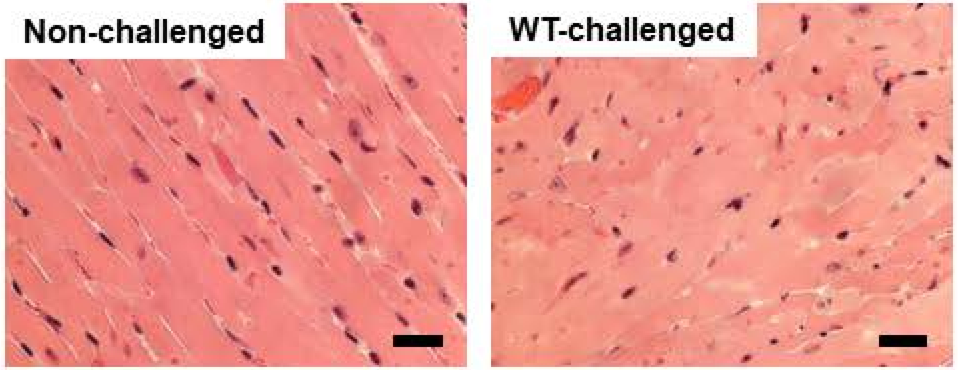

Supplementary Fig. 13: Analysis of mice inoculated with DKO parasites and treated with dexamethasone. a) explantation analysis of organs harvested from mice inoculated with DKO *T. cruzi* and treated with dexamethasone (for 30 days) at day 365 post-inoculation (non-challenged) (see Fig. 3) and those multi-inoculated with DKO parasites and then challenged with WT parasites (WT-challenged)(see Fig. 8). The numerator refers to the number of animals with out-growth of parasites from the indicated tissue in culture medium and the denominator is the total number of animals analyzed. b) Representative histopathological analysis of heart tissue of non-challenged and WT-challenged mice and treated with dexamethasone showing the absence of parasite nests. Scale bar = 25 μm.

PCR analysis of Cyp19 knockout parasite lines (Fig.1B)

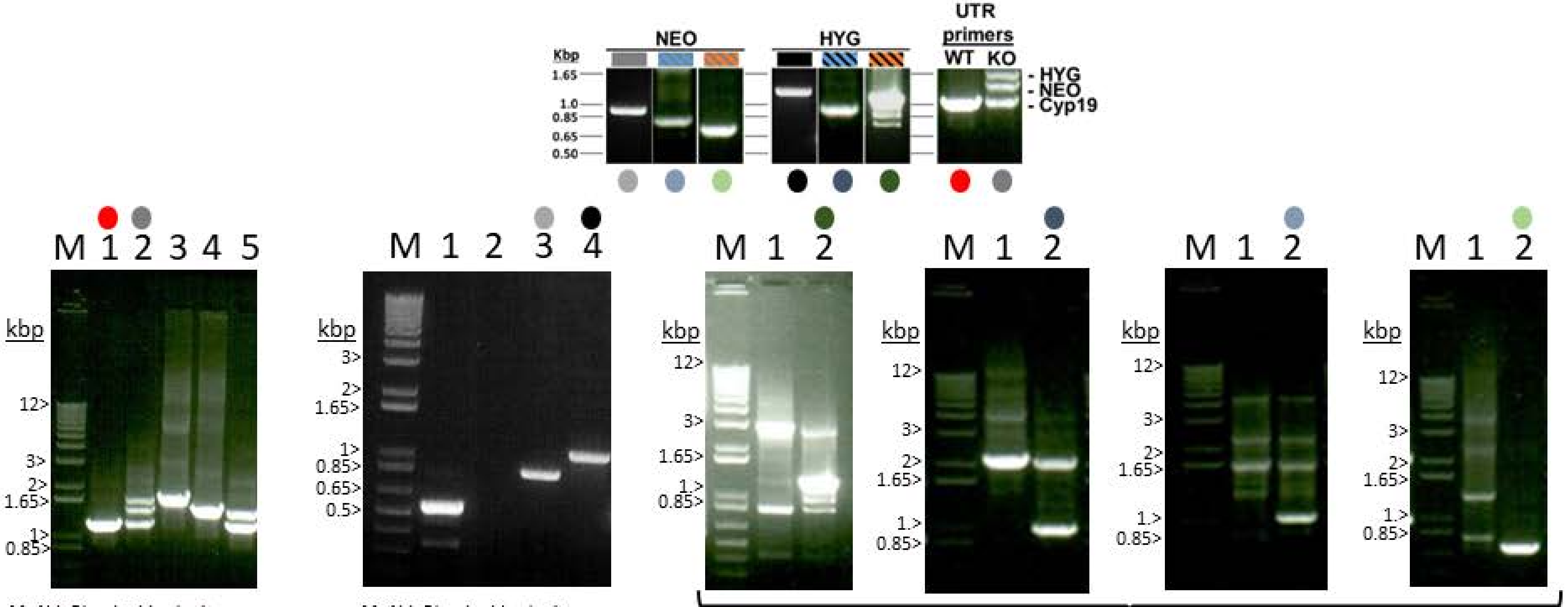

M: 1kb Plus Ladder-Invitrogen
1: Genomic DNA from T. cruzi (WT)
2: Genomic DNA from T. cruzi (DKO)
3: Control DNA 5'-Hyg-3'
4: Control DNA 5'-Neo-3'
5: SKO-NEO-genomic DNA

Primers used: 5'UTR-F + 3'UTR-R

M: 1kb Plus Ladder-Invitrogen
1: Genomic DNA from T. cruzi (WT)
2: No DNA
3: Genomic DNA from T. cruzi (DKO)
4: Genomic DNA from T. cruzi (DKO)

Primers used:
1) 5'UTR-F + 3'UTR-R
2) 5'UTR-F + 3'UTR-R
3) 5'-Neo-F + 3'-Neo-R
4) 5'-Hyg-F + 3'-Hyg-R

M: 1kb Plus Ladder-Invitrogen
1: Genomic DNA from T. cruzi (WT)
2: Genomic DNA from T. cruzi (DKO)

Primers used:
5' flank (blue)-F +
3' Hyg internal-R

Primers used:
Hyg internal-F +
3' flank-R

Primers used:
5' flank-F +
Neo internal-R

Primers used:
Neo internal-F +
3'flank)-R

Supplementary Fig. 14: Source data for the analysis of knock-out parasite lines using PCR for Fig.1B. The indicated purified genomic DNA was used for PCR analysis with the indicated primer pairs and subjected to agarose gel electrophoresis. Colored dots indicate corresponding lanes of composite figure and source data.

Confirmation of Cyp19 KO from *T. cruzi* by western blot (Fig.1C)

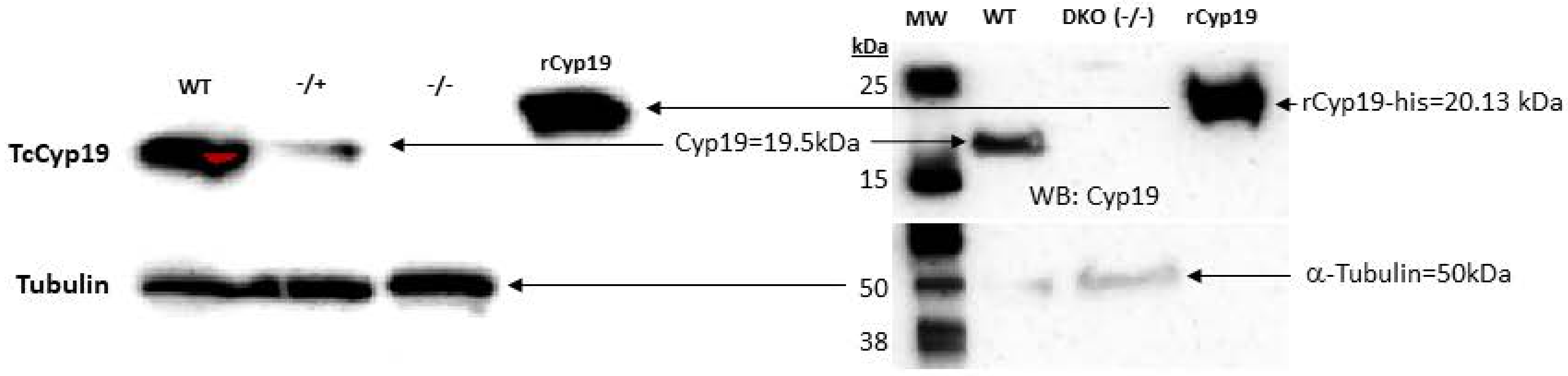

Gel with all 3 parasite samples (Fig.1C)

Gels with MW markers verifying MW of Cyp19 and α–tubulin

Supplementary Fig. 15: Demonstration of molecular size of proteins bands in Fig. 1C. Wt Cyp19 is 177 residues (19.47 kDa) and α-tubulin is 50 kDa. Recombinant Cyp19 (rCyp19, which contains a 6xhis-tag, is 20.13 kDa. These sizes are shown on control blots in the right panel and correspond to those on the left panel using rCyp19 as a reference.

Confirmation of PCR analysis of cardiac tissue of infected mice (Supplementary Fig. 7)

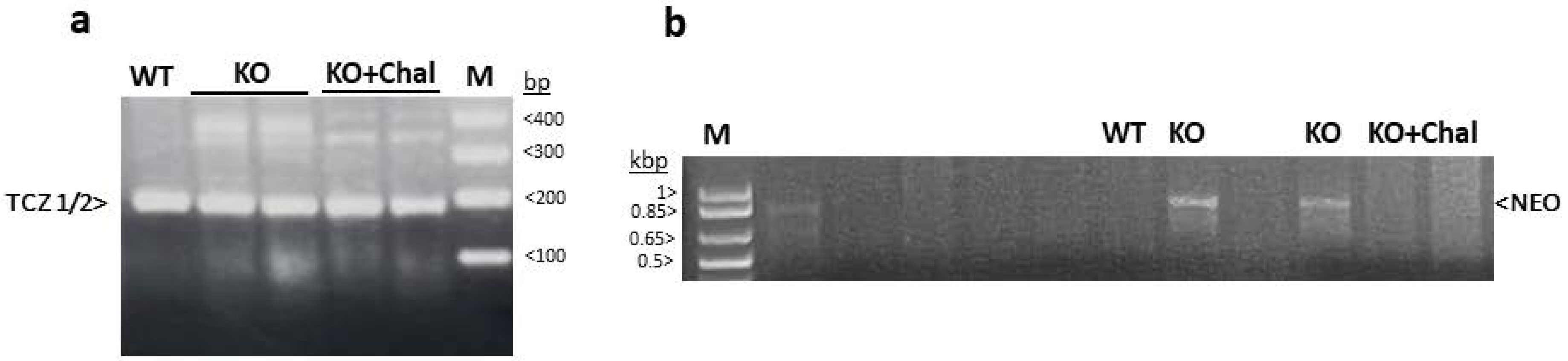

Supplementary Fig. 16: Source data for Supplementary Fig. 7 (PCR analysis of cardiac tissue of infected mice). a) PCR bands for the *T. cruzi* specific TCZ gene using TCZ 1 and 2 primers (182bp). b) PCR bands specific for the neomycin phosphotransferase gene (NEO) (855bp). Indicated lane designations correspond to the composite data in Supplementary Fig. 7. M: 1kb Plus Ladder-Invitrogen.
