# Supplementary material for: Immunization with a Trypanosoma cruzi cyclophilin-19 deletion mutant protects against acute Chagas disease in mice
Source: NPJ Vaccines. 2023 Apr 25;8:63. doi: 10.1038/s41541-023-00647-5 (PMC10130101; doi:10.1038/s41541-023-00647-5)
Supplement: Supplementary file 2 — REPORTING SUMMARY [file 41541_2023_647_MOESM2_ESM.pdf]

nature portfolio

Corresponding author(s): McGwire, B. S.

Last updated by author(s): Mar 14, 2023

Reporting Summary

Nature Portfolio wishes to improve the reproducibility of the work that we publish. This form provides structure for consistency and transparency in reporting. For further information on Nature Portfolio policies, see our Editorial Policies and the Editorial Policy Checklist.

Statistics

For all statistical analyses, confirm that the following items are present in the figure legend, table legend, main text, or Methods section.

n/a	Confirmed	
☐	☒	The exact sample size (*n*) for each experimental group/condition, given as a discrete number and unit of measurement
☐	☒	A statement on whether measurements were taken from distinct samples or whether the same sample was measured repeatedly
☐	☒	The statistical test(s) used AND whether they are one- or two-sided<br>*Only common tests should be described solely by name; describe more complex techniques in the Methods section.*
☒	☐	A description of all covariates tested
☒	☐	A description of any assumptions or corrections, such as tests of normality and adjustment for multiple comparisons
☐	☒	A full description of the statistical parameters including central tendency (e.g. means) or other basic estimates (e.g. regression coefficient) AND variation (e.g. standard deviation) or associated estimates of uncertainty (e.g. confidence intervals)
☐	☒	For null hypothesis testing, the test statistic (e.g. *F*, *t*, *r*) with confidence intervals, effect sizes, degrees of freedom and *P* value noted<br>*Give P values as exact values whenever suitable.*
☒	☐	For Bayesian analysis, information on the choice of priors and Markov chain Monte Carlo settings
☒	☐	For hierarchical and complex designs, identification of the appropriate level for tests and full reporting of outcomes
☒	☐	Estimates of effect sizes (e.g. Cohen's *d*, Pearson's *r*), indicating how they were calculated

Our web collection on statistics for biologists contains articles on many of the points above.

Software and code

Policy information about availability of computer code

Data collection	For ELISA: Softmax Pro Software (Molecular Devices LLC, Sunnyvale, CA); For Flow cytometry (Flowjo version 10 from Tree Star, Ashland, OR); For DNA sequencing: Geneious software (Biomatters, INC, San Diego Ca).
Data analysis	Using above software and Microsoft Excel-2019 and Graphpad/Prism 9 for statistical analysis.

For manuscripts utilizing custom algorithms or software that are central to the research but not yet described in published literature, software must be made available to editors and reviewers. We strongly encourage code deposition in a community repository (e.g. GitHub). See the Nature Portfolio guidelines for submitting code & software for further information.

Data

Policy information about availability of data

All manuscripts must include a data availability statement. This statement should provide the following information, where applicable:

- Accession codes, unique identifiers, or web links for publicly available datasets
- A description of any restrictions on data availability
- For clinical datasets or third party data, please ensure that the statement adheres to our policy

The authors confirm that the data supporting the findings of this study are available within the article and its supplementary materials. Use of the DKO strain is patent protected through the Ohio State Innovation Foundation (patent #11110132).

Human research participants

Policy information about studies involving human research participants and Sex and Gender in Research.

Reporting on sex and gender	We did not use human participants in this study.
Population characteristics	*Describe the covariate-relevant population characteristics of the human research participants (e.g. age, genotypic information, past and current diagnosis and treatment categories). If you filled out the behavioural & social sciences study design questions and have nothing to add here, write "See above."*
Recruitment	*Describe how participants were recruited. Outline any potential self-selection bias or other biases that may be present and how these are likely to impact results.*
Ethics oversight	*Identify the organization(s) that approved the study protocol.*

Note that full information on the approval of the study protocol must also be provided in the manuscript.

Field-specific reporting

Please select the one below that is the best fit for your research. If you are not sure, read the appropriate sections before making your selection.

☒ Life sciences ☐ Behavioural & social sciences ☐ Ecological, evolutionary & environmental sciences

For a reference copy of the document with all sections, see nature.com/documents/nr-reporting-summary-flat.pdf

Life sciences study design

All studies must disclose on these points even when the disclosure is negative.

Sample size	For studies in mice, samples sizes of 5 per group/per experiment. This is the standard sample sizes used for studies in our lab. Replicate experiments were carried out when feasible and total sample sizes from all experiments were combined where appropriate. For sampling of each animal. In vitro experiments (e.g., infections assays in cell lines, growth curves, etc) were generally performed 3-5 times to confirm results.
Data exclusions	No data was excluded.
Replication	Experiments were performed multiple times to demonstrate reproducibility.
Randomization	For inoculation studies, mice were randomly selected for each experimental group. Samples (blood, tissue, etc) were collected from individual subsets of mice at various points and these mice were selected randomly.
Blinding	Investigators were not blinded to group allocation, since experiments were performed with and data analyzed on samples collected from designated groups.

Reporting for specific materials, systems and methods

We require information from authors about some types of materials, experimental systems and methods used in many studies. Here, indicate whether each material, system or method listed is relevant to your study. If you are not sure if a list item applies to your research, read the appropriate section before selecting a response.

Materials & experimental systems

n/a	Involved in the study	
☐	☒	Antibodies
☐	☒	Eukaryotic cell lines
☒	☐	Palaeontology and archaeology
☐	☒	Animals and other organisms
☒	☐	Clinical data
☒	☐	Dual use research of concern

Methods

n/a	Involved in the study	
☒	☐	ChIP-seq
☐	☒	Flow cytometry
☒	☐	MRI-based neuroimaging

Antibodies

Antibodies used	Polyclonal anti-cyclophilin Abs were produced in our lab using recombinant expressed protein and prepared commercially to synthetic peptides. Secondary antibodies were purchased commercially.
Validation	Primary Abs were validated using recombinant protein (expressed from sequenced verified cloned genes) as well as protein lysates from parasites.

Eukaryotic cell lines

Policy information about cell lines and Sex and Gender in Research

Cell line source(s)	Parasite cell lines have been propagated in my lab for decades; they were originally acquired from my mentor David Engman (previously at Northwestern Univ). H9C2 (rat heart myoblasts) were purchased originally from the ATCC.
Authentication	Cell lines have not been authenticated.
Mycoplasma contamination	Cell lines have not been screened for mycoplasma contamination.
Commonly misidentified lines (See ICLAC register)	No misidentified lines were used in our experiments.

Animals and other research organisms

Policy information about studies involving animals; ARRIVE guidelines recommended for reporting animal research, and Sex and Gender in Research

Laboratory animals	Mice used in these studies: Male AJ mice 4-6 weeks of age; male STAT1-/-(BALB/c), and STAT4-/-(BALB/c) 4-6 weeks of age.
Wild animals	No wild animals were used in these studies.
Reporting on sex	Sex was not considered for this study design.
Field-collected samples	Samples were not collected from the field.
Ethics oversight	All animal experimental protocols were approved by the Ethical Committee for Animal Research of the Ohio State University.

Note that full information on the approval of the study protocol must also be provided in the manuscript.

Flow Cytometry

Plots

Confirm that:

☒ The axis labels state the marker and fluorochrome used (e.g. CD4-FITC).

☒ The axis scales are clearly visible. Include numbers along axes only for bottom left plot of group (a 'group' is an analysis of identical markers).

☒ All plots are contour plots with outliers or pseudocolor plots.

☒ A numerical value for number of cells or percentage (with statistics) is provided.

Methodology

Sample preparation	The presence of trypanolytic antibodies in serum was assessed using carboxyfluorescein succinimidyl ester (CFSE) labeled parasites in a flow cytometry-based system. Wildtype RHM-derived trypomastigotes were labeled with PBS containing CFSE for 30 mins at RT making them fluorescent and detectable in the FITC channel. Labeled parasites were incubated with serial diluted immune serum followed by human complement for 4 hrs. Lysis of parasites was measured by diminished CFSE fluorescence in flow cytometry. Control included parasites incubated in non-immune serum + complement, complement alone and heat-inactivated complement.
Instrument	FACS Celesta, BD Biosciences
Software	Flowjo ver 10 (Tree Star INC, Ashland OR)
Cell population abundance	Pure populations of cultured cells were analyzed before and after treatment. Post sort fractions of were sampled microscopically to verify presence of live and dead parasites.
Gating strategy	Pure populations of cultured cells were analyzed before and after treatment. Individual events were determined by diminished fluorescence (parasites killed) using the FITC channel and these were were quantitated and expressed as a % of

the total population. Positive controls of stained cells determined the gate to which individual reactions were referenced. Our gating strategy is shown in the supplementary material.

☒ Tick this box to confirm that a figure exemplifying the gating strategy is provided in the Supplementary Information.
